# Evaluating the reach and equity of public spaces in Mexico City using mobility data: the case of the Utopías in Iztapalapa

**DOI:** 10.3389/fdata.2026.1942458

**Published:** 2026-09-23

**Authors:** Ollin D. Langle-Chimal, Natalia Cadavid-Aguilar, Alberto Meouchi-Vélez, Roberto Ponce-López

**Affiliations:** 1 Department of Civil and Environmental Engineering, University of California, Berkeley, Berkeley, CA, United States; 2 Tecnologico de Monterrey, Center for the Future of Cities, Mexico City, Mexico; 3 Tecnologico de Monterrey, School of Government and Public Transformation, Monterrey, Mexico

**Keywords:** accessibility, human mobility, public space, public transport, urban parks, urban segregation

## Abstract

Cities invest in social infrastructure to improve well-being, but the benefits depend on who can use these spaces and who actually does. Access is therefore not only a matter of whether people live close enough to reach a facility, but also of who travels to it and from how far. We examine this realized access using high-resolution human mobility data from Location-Based Services (LBS) for the Utopías, a network of thirteen community centers offering free recreation, culture, and care in Iztapalapa, an underserved borough of 1.8 million residents in eastern Mexico City. We combine six months of mobility traces with public transit data (GTFS), a georeferenced crime registry, and a catalog of facility activities to identify the factors associated with each site's visitors. Three factors capture distinct dimensions of reach. Transit connectivity tracks the number of visitors a site draws. Facilities with more distinctive activities, such as a planetarium or a dinosaur park, show a greater increase in visitor travel distance at weekends. By contrast, a higher share of violent crime in the surrounding area is associated with more localized and lower-income visitor catchments. Together, these findings show that the reach of social infrastructure varies not only with proximity, but also with transport, the distinctiveness of what facilities offer, and local safety.

## 1 Introduction

Public space is one of the levers a city government has for redistributing well-being. People express measurably greater happiness while at an urban park than elsewhere in the same city (Schwartz et al., 2022), and access to green space has been shown to weaken the relationship between income and mortality (Mitchell and Popham, 2008). Placing neighborhood facilities in underserved areas, whether a park, a swimming pool, a library or a free clinic, can shift the daily geography of opportunity. In Medellín, where library parks and other facilities were built alongside new cable-car lines under the banner of social urbanism, violence fell further than in otherwise similar neighborhoods (Cerdá et al., 2012). But the value of these facilities is realized only through use, and physical proximity does not guarantee visitation, particularly in low-income areas (Cohen et al., 2016; Linnell et al., 2023). Use depends on whether residents can reach them (Wolch et al., 2014; Boone et al., 2009), find the time to be there (Sánchez De Madariaga and Arvizu Machado, 2025), and feel safe enough to stay (Foster et al., 2014).

Measuring whether a facility is actually used, and by whom, has been difficult at the scale of a whole borough. Surveys that stop visitors on site are costly (McKenzie et al., 2006), surveys that ask people to recall past visits are slow and systematically miss the short trips a neighborhood facility generates (Sammer et al., 2018), and a headcount at the entrance counts visitors without describing them. Mobility data derived from location-based services (LBS) now make it possible to observe visitation to specific places at a spatial and temporal resolution that surveys cannot match (Barbosa et al., 2018; Blondel et al., 2015). We bring this kind of data to the Utopías of Iztapalapa, a large social infrastructure program in Mexico City that is still taking shape.

The Utopías are a network of community centers and the main social program of Iztapalapa, a borough on the eastern periphery of Mexico City. The local government began building the centers in 2019, to offer recreational and educational activities, green and open space, and care services to the low-income population that predominates in the borough (Zaragoza Ramírez, 2024; Colin Colin et al., 2024). Iztapalapa is home to nearly 1.8 million people, more than any other borough, and has long combined high population density with little green space and consolidated irregular settlements (Delgadillo, 2016; Monkkonen et al., 2021; Ruiz-Rivera et al., 2016). It has 5.4 square meters of green space per resident, roughly a third of the 15.4 in the best served borough (Núñez, 2021), and 43.9% of its residents were living in poverty in 2020 (CONEVAL, 2021). These deficits weigh most heavily on women, who carry a disproportionate share of care responsibilities and make many short trips close to home to meet them, the kind of travel that nearby facilities are well suited to serve (Sánchez De Madariaga and Arvizu Machado, 2025). Several Utopías respond to this directly, pairing their recreation offering with women's physical and mental health programs and other support services.

Each site is built around a different mixture of facilities, ranging from swimming pools and skateparks to climbing walls, science exhibits and community kitchens. A few carry a signature attraction, such as a planetarium or a dinosaur park. Existing accounts of the program set out what was built and what it was meant to do, but they do not measure who uses it (Zaragoza Ramírez, 2024; Colin Colin et al., 2024). That leaves two questions unanswered. Who does the network actually reach, and why do some sites attract substantially more users than others?

The program is intended to lower the cost of reaching opportunities that the eastern periphery has lacked. That places the question within the accessibility literature, where the value of a facility depends on how many people can reach it and at what cost (Hansen, 1959). Parks and public amenities have been studied this way, with distance, income and transport infrastructure all associated with who uses them (Wolch et al., 2014; Boone et al., 2009; Sister et al., 2010; Talen, 2003), including in Latin American cities (Bocarejo and Oviedo, 2012; Vecchio et al., 2020). Most of these studies measure potential access, the population that could reach a site. Facility-led investment in Latin American peripheries has been evaluated mainly for Medellín, through resident perception surveys or the position of each site in the street network, and critics note that such programs have often been replicated with more attention to the visibility of the buildings than to their use (Holmes and Gutiérrez de Piñeres, 2014; Goodship and Capille, 2017; Whitney and Sotomayor, 2025). Aggregated traces from mobile devices let us observe revealed visitation instead, and have been used to characterize individual movement (González et al., 2008; Song et al., 2010), to measure the wealth mix that people experience across the places they visit (Moro et al., 2021; Athey et al., 2021; Wang et al., 2018; Xu et al., 2025), to read urban structure from commuting flows (Louail et al., 2015; Zhao et al., 2024; Liao et al., 2025), and to study access to essential services where survey data are scarce (Melikov et al., 2021). Despite their broad use, data of this kind carry a sampling bias toward younger and wealthier users (Cabrera and Rowe, 2026).

The Utopías offer an unusually well-controlled setting for this analysis. Because all thirteen sites sit within a single borough, the broad contextual differences that usually confound studies of park equity are largely held constant, so the variation we study is variation between the sites themselves. They differ visibly in what they provide, from standard recreation packages to attractions that exist nowhere else in the network, which gives us within-borough variation in the distinctiveness of the offer. They stand in very different transit environments, which gives us variation in accessibility to public transport. And the neighborhoods around them differ measurably in crime, in both volume and composition, which lets us ask how surrounding safety relates to reach. Because the program is expanding to other parts of the city, the questions of what use depends on and how to measure it from mobility traces and open administrative data are immediately useful for policy, here and in any city weighing the same kind of investment.

Our aim is to measure who the Utopías actually reach, and to identify what separates the sites that draw from across the metropolitan area from those serving their immediate surroundings. We do this for all 13 sites over a 6-month window, using mobility data alongside open official data. We measure realized rather than potential access, using mobility traces to observe who visits each site, when they visit, where they come from, and how far they travel. Transport access, the distinctiveness of the service offer and the surrounding crime track different dimensions of reach. Transport access is associated with visitor volume, distinctiveness with how far visitors travel, particularly on weekends, and the violent share of crime with the socioeconomic composition of visitors. Our design is a cross-section of 13 sites observed in a single time period, so we interpret these relationships as associations rather than causal effects. Furthermore, the mobility data under-represent the poorest residents, the very population that the program intends to serve, so our counts likely understate how many of them the network reaches.

## 2 Materials and methods

Our analysis has three stages. We first build the visit record, detecting stops in the raw LBS traces, assigning each device a home location and a residential wealth value, and reconstructing the perimeter of each site so that a stop can be attributed to a Utopía. We then characterize every site along three axes that a municipal government can observe before a facility is built, the distinctiveness of the activities on offer, the public transport available within a one-kilometer buffer, and the volume and composition of surrounding crime. Finally we aggregate the visit record into per-site outcomes, among them the count of unique visitors, the distances traveled, the weekend distance premium, meaning the extra distance weekend visitors travel, and the wealth mix of the catchment, and relate the three site axes to those outcomes.

### 2.1 Study area and data sources

We study the thirteen Utopías built by the borough of Iztapalapa: Olini, Meyehualco, Teotongo, Tezontli, La Cascada, Quetzalcoatl, Libertad, Atzintli, Ixtapalcalli, Cuauhtlicalli, Papalotl, Tecoloxtitlan, and Barco, all located in the eastern periphery of Mexico City. The borough covers roughly 113 km^2^ and is home to about 1.8 million people, most of them in the lower half of the metropolitan wealth distribution. Visitor homes, by contrast, are detected across the Zona Metropolitana del Valle de México (ZMVM), comprising the sixteen boroughs of the city, the neighboring municipalities of the State of Mexico, and one municipality in Hidalgo, so origins are measured over the whole region rather than the borough alone. We combine five open or licensed sources: anonymized location-based services (LBS) traces from a single commercial provider, a census-derived map of residential wealth for the metropolitan area (Lucchini et al., 2025), the city's GTFS public-transport feed, the open crime registry of the Fiscalía General de Justicia (FGJ), the Mexico City prosecutor's office, and a hand-built catalog of the activities offered at each site. The LBS extract spans late September 2023 to the end of March 2024, roughly 6 months.

### 2.2 Mobility data: stop detection

The raw LBS feed is a stream of device pings, each consisting of a latitude, longitude and timestamp. We process it into stop events using the same pipeline as earlier work on Mexico City mobility (Lucchini et al., 2025); the implementation is openly available and archived (Langle-Chimal, 2026). For a device with ping sequence {(**x**_*t*_, *t*)} we first detect *stay events*: maximal runs of consecutive pings that remain within a radius *r*_0_ = 20 m of their running centroid and span at least τ_0_ = 5 min, which we treat as periods during which the user was stationary. We then cluster the stay events of each device with DBSCAN (ϵ = 20 m, minimum cluster size of 2) so that repeated stays at the same place collapse into a single stop location, while places that a device visits only once are retained as individual stop locations. The output is, for every device, a set of stop locations with the days and local hours at which each was visited.

### 2.3 Home labeling and residential wealth

We assign a home label to the stop cluster a device visits most often during residential hours, defined as all hours on weekends and 19:00 to 08:00 on weekdays in local time. Candidate clusters are restricted to those the device visits on at least 14 distinct days over the observation window, and we exclude devices with no qualifying cluster. Each detected home is then matched to the metropolitan wealth map, which partitions the ZMVM into 5,648 residential cells. The wealth value of a residential cell is the first principal component of a set of census socioeconomic variables (income, schooling, housing quality and household assets), rescaled to the unit interval so that higher values denote greater wealth (Lucchini et al., 2025); we write *W*_*i*_ for the wealth of the cell containing home *i*. We compute the quartile cutpoints of *W* once on the metropolitan distribution and apply them to every site, so that visitor wealth is expressed on a common city-wide scale rather than relative to each site's own catchment. Each home also falls within a known state, municipality or borough, and census block (manzana), which we use to attribute every visitor to their origin.

### 2.4 Utopía perimeter reconstruction

No official source delineates the boundaries of the Utopías. We therefore reconstructed their perimeters using the publicly available dataset of WiFi access points installed at Utopía facilities,^1^ whose access-point coordinates fall inside each site and provide a reliable anchor. Working from these anchors in ArcGIS Pro, we digitized a polygon around the visible extent of each Utopía using high-resolution satellite imagery, tracing perimeter walls, fences, and built edges where they were legible. Because several sites are composed of multiple polygons, we merged them into a single geometry per site. We then cross-checked every boundary against the program's official site descriptions^2^ and imagery, adjusting polygons where the two sources disagreed. One site, Ixtapalcalli, was absent from the WiFi dataset; for it we relied on the satellite imagery and the program's official description alone. The thirteen sites range from under a hectare at the smallest to several tens of hectares at Olini, the largest.

### 2.5 Activities on offer and the distinctiveness index

We compiled a catalog of the facilities and activities at each site from the borough's published descriptions, grouped the individual entries into thematic categories by manual inspection, and collapsed the catalog to one record per site and category, so that a site's offer is described by the distinct service categories it provides. We further aggregate these categories into twelve domains (aquatics, team sports and athletics, combat and extreme sports, racquet and court sports, fitness, performing arts and culture, education and technology, nature and biodiversity, wellness and therapy, social services, general recreation, and landmark attractions). Let *N* = 13 be the number of sites and *n*_*c*_ the number of sites that offer category *c*. Each category receives a rarity score ρ_*c*_ = 1−*n*_*c*_/*N*, which is high for offerings that are unique in the network, and an infrastructure-intensity score ι_*c*_∈{1, 2, 3} that separates standard services (ι_*c*_ = 1; an outdoor gym, an arts workshop), specialized facilities (ι_*c*_ = 2; a boxing gym, a skate park) and capital-intensive signature attractions (ι_*c*_ = 3; a wave pool, a dinosaur park). The per-category distinctiveness weight is the product *w*_*c*_ = ρ_*c*_ι_*c*_, and the site-level distinctiveness index is the mean of this weight over the categories a site offers,


$$\begin{array}{l} D_{a}=\frac{1}{\left|C_{a}\right|}\underset{c\in C_{a}}{\sum }\rho_{c}\iota_{c}, \end{array}$$ (1)

where the sum runs over *C*_*a*_, the distinct service categories present at site *a*, and |*C*_*a*_| is how many of them the site offers, a count we call the site's facility count. *D*_*a*_ is high for sites that pair rare offerings with capital-intensive infrastructure and low for sites built around standard, easily replicated services; among our thirteen sites Barco scores highest and La Cascada lowest. For the domain-level analysis we additionally use the count of a site's categories in each of the twelve domains, and in the composite below we use the facility count |*C*_*a*_| as a measure of the breadth of the offer. The categories offered at each site, and the rarity, infrastructure-intensity score and distinctiveness weight of every category, are listed in full in the Supplementary material. Robustness checks reported there show that the association between *D*_*a*_ and the weekend distance premium holds under eight alternative operational definitions of Equation 1, covering how rarity and infrastructure intensity are combined, how rarity is scaled, and whether a site's score is the mean, the sum or the maximum over its categories. The chosen aggregation is sensitive to sites with fewer and rarer offerings, which receive a high score.

### 2.6 Public transport accessibility

We construct the transit profile of each Utopía using the General Transit Feed Specification (GTFS) feed published by the city's Secretaría de Movilidad, covering the metro, Metrobús, trolebús, RTP, light rail, suburban rail, and the colectivo network. The unit of analysis is a one-kilometer buffer *B*_*a*_ drawn around the site perimeter. Using the perimeter rather than the centroid prevents underestimating access at larger facilities, where a centroid-based radius would fall largely within the park's boundaries. Within *B*_*a*_, we measure the number of stops, distinct routes *R*_*a*_, the number of transit modes *k*_*a*_, and total scheduled weekday and weekend trips (*V*_*a*_).

We also construct binary indicators $1_{a}^{\text{metro}}\in \left\{0,1\right\}$ and $1_{a}^{\text{BRT}}\in \left\{0,1\right\}$ indicating the presence of at least one metro or BRT stop within *B*_*a*_. Letting $\tilde{x}$ denote the min-max rescaling of variable *x* to the interval [0, 1] across all thirteen sites, the composite transit accessibility score is defined as:


$$\begin{array}{l} T_{a}=0.30\tilde{\log\left(1+R_{a}\right)}+0.30\tilde{\log\left(1+V_{a}\right)}+0.201_{a}^{\text{metro}}+0.101_{a}^{\text{BRT}}\\ \;+0.10\tilde{k_{a}}. \end{array}$$ (2)

The weights in Equation 2 are defined a priori to prioritize route diversity and trip frequency while incorporating modest premiums for rapid transit modes. Robustness checks detailed in the Supplementary material confirm that the score's association with visitor volume remains consistent under alternative weighting schemes (equal-weighted, rail-heavy, and supply-only parameterizations) and under an unsupervised principal component analysis (PCA) of the five inputs. Across all specifications, route diversity and weekday trip frequency provide the primary predictive signal.

### 2.7 Crime data processing

We take crime records from the FGJ open data portal for 2023 and 2024. Each record carries the coordinates of the incident, the offense label, and two dates: the date the report was filed (*fecha_inicio*) and the date the incident occurred (*fecha_hecho*). Because the filing date can post-date the incident by years, we use *fecha_hecho* for any temporal series and clip to the active reporting window so that retrospectively filed incidents do not stretch the series. We keep incidents with valid coordinates inside the ZMVM bounding box and assign each to a site if it falls within that site's one-kilometer perimeter buffer *B*_*a*_. The prosecutor's free-text offense labels are too granular for cross-site comparison, so we map them onto fifteen analytic categories (intentional homicide, vehicle theft, pedestrian robbery on the public road, microbus, metro and taxi passenger robbery, business robbery with violence, home burglary with violence, ATM customer robbery, delivery-worker and cargo-transporter robbery, firearm injury, kidnapping, rape, and low-impact crime) using a hand-encoded lookup table, which we validate against the registry's own *categoria_delito* field, excluding a non-criminal residual. For each site, we record the total count $n_{a}^{\text{crime}}$, the density $\kappa_{a}=n_{a}^{\text{crime}}/|B_{a}|$ per square kilometer of buffer, the per-category counts, and the violent share (Equation 3),


$$\begin{array}{l} \upsilon_{a}=\frac{n_{a}^{\text{viol}}}{n_{a}^{\text{crime}}}, \end{array}$$ (3)

where $n_{a}^{\text{viol}}$ counts incidents in the violent categories (homicide, firearm injury, kidnapping, rape, and the violent variants of robbery). We use this categorical breakdown to ask whether one kind of crime, rather than overall volume, tracks the visit response, since aggregate counts are dominated by a few high-volume categories that may not be the most behaviorally relevant. Survey-based measures of perceived insecurity are available only at the borough level, where Iztapalapa registers among the highest in the city (INEGI, 2024); we therefore work with georeferenced recorded incidents, which resolve the within-borough, site-level variation that a borough-wide perception figure cannot.

### 2.8 Per-site outcomes, accessibility and place-level segregation

We work at the level of (visitor, day, and Utopía) and aggregate up to per-site outcomes. These outcomes include: the count of unique visitors, our headline measure, preferred over total stops because total stops are dominated by a handful of high-frequency users; the recurrence rate, the share of visitors who return at least once; the mean and median great-circle distance from home to site; the share of visits on weekends; and the weekend distance premium, defined as the difference in mean distance from home to site between weekend and weekday visitors.

We summarize how accessible each site is to the surrounding population with a distance-weighted accessibility index (Equation 4),


$$\begin{array}{l} A_{a}=\underset{j}{\sum }\exp(-(d_{aj}/d_{0}{)}^{2}), \end{array}$$ (4)

where *j* runs over the cells of the metropolitan wealth map, *d*_*aj*_ is the distance from site *a* to cell *j*, and *d*_0_ = 5 km is a Gaussian bandwidth. The index is high for sites placed centrally within the borough, where a large surrounding population can reach them easily.

For the socioeconomic composition of visitors we use the place-level segregation index *S*_*a*_ of Moro et al. (2021). Let τ_*aq*_ be the share of unique visitors of Utopía *a* whose home falls in metropolitan wealth quartile *q*. Then


$$\begin{array}{l} S_{a}=\frac{2}{3}\overset{4}{\underset{q=1}{\sum }}|\tau_{aq}-0.25|, \end{array}$$ (5)

bounded between zero, when a site draws an even mix across the four quartiles, and one, when it draws entirely from a single quartile. In the Moro framework *S*_*a*_ measures the wealth mix a place attracts, as distinct from the individual-level index *S*_*i*_ that measures the mix a person encounters across all the places they visit; our question is about destinations, so we use *S*_*a*_. We prefer it to a Gini coefficient because it is bounded, easy to interpret, and directly comparable to a growing literature on experienced segregation (Moro et al., 2021; Athey et al., 2021; Xu et al., 2025).

### 2.9 The combined accessibility index

We combine the three profiles into a single composite score, both to summarize them in one ordering and to test whether they carry a common signal. Because the underlying measures are on different scales, we first standardize each by computing its z-score across the thirteen sites, so that each measure is expressed in standard deviations from the network average. We then form one axis per profile by averaging the z-scores of its measures. The services axis averages the z-scores of the distinctiveness index and the facility count, $z_{a}^{\text{S}}=\frac{1}{2}[z\left(D_{a}\right)+z\left(\left|C_{a}\right|\right)]$; the transit axis is simply the standardized transit score, $z_{a}^{\text{T}}=z\left(T_{a}\right)$, since that score already integrates routes, trips, metro, BRT and modes; and the crime axis averages the z-scores of crime density and the violent share, $z_{a}^{\text{C}}=\frac{1}{2}[z\left(\kappa_{a}\right)+z\left(\upsilon_{a}\right)]$. The composite gives the three axes equal weight and subtracts the crime axis, so that higher values always indicate better accessibility and reach (Equation 6),


$$\begin{array}{l} K_{a}=\frac{1}{3}(z_{a}^{\text{S}}+z_{a}^{\text{T}}-z_{a}^{\text{C}}). \end{array}$$ (6)

We report rankings under three alternative weight schemes in the Supplementary material. The composite is deliberately simple. It is intended to support comparative ranking rather than represent a welfare function, which would require survey-based preference weights that are currently unavailable. Combining dimensions into one score is standard practice for the agencies that would use this kind of measure. The multidimensional poverty measures now used across Latin America work the same way, reporting each deprivation on its own and also adding them into a single number. Ours is a simpler construction, and it does not replace reading the three axes separately, which is where the distinct mechanisms are visible. What it provides is one ordering of candidate sites that carries more than one dimension at a time, and its value would grow as further dimensions, such as service quality, staffing or opening hours, are added to it.

### 2.10 Statistical analysis

Throughout, we report Pearson correlations with two-sided permutation tests (up to 10, 000 permutations) and bootstrap 95% confidence intervals (up to 5, 000 resamples). For the heavily skewed visit counts, we compute correlations using the base-ten logarithm. Where we separate the three axes, we use partial correlations based on linear residuals and fit a standardized linear regression of log visits on the axes. With thirteen sites, these tests have limited statistical power, so we interpret associations with *p*-values between 0.05 and 0.10 as suggestive rather than conclusive, and we treat every relationship as associational rather than causal.

## 3 Results

### 3.1 Spatial distribution of the network and visitor origins

The thirteen Utopías are distributed across Iztapalapa, yet the network is characterized by a highly uneven distribution of visitation (Figures 1a, b). The two largest sites, Olini and Meyehualco, concentrate the vast majority of mobility flows, accounting for roughly 2,700 and 1,200 unique visitors over the observation period, collectively representing more than four in five of all recorded visits. The remaining facilities operate on a different scale, with Teotongo the only intermediate node with over three hundred unique visitors, while the other ten draw only a few dozen or a hundred. Despite their lower visitor volumes, these neighborhood-scale facilities maintain consistent day-to-day use. Their active-day counts reveal a far more stable baseline of daily use, supported by a small but highly consistent cohort of recurring local visitors. The primary axis of differentiation across the network is therefore not whether a site is visited, but rather the spatial scale of its geographic catchment.

**Figure 1:**
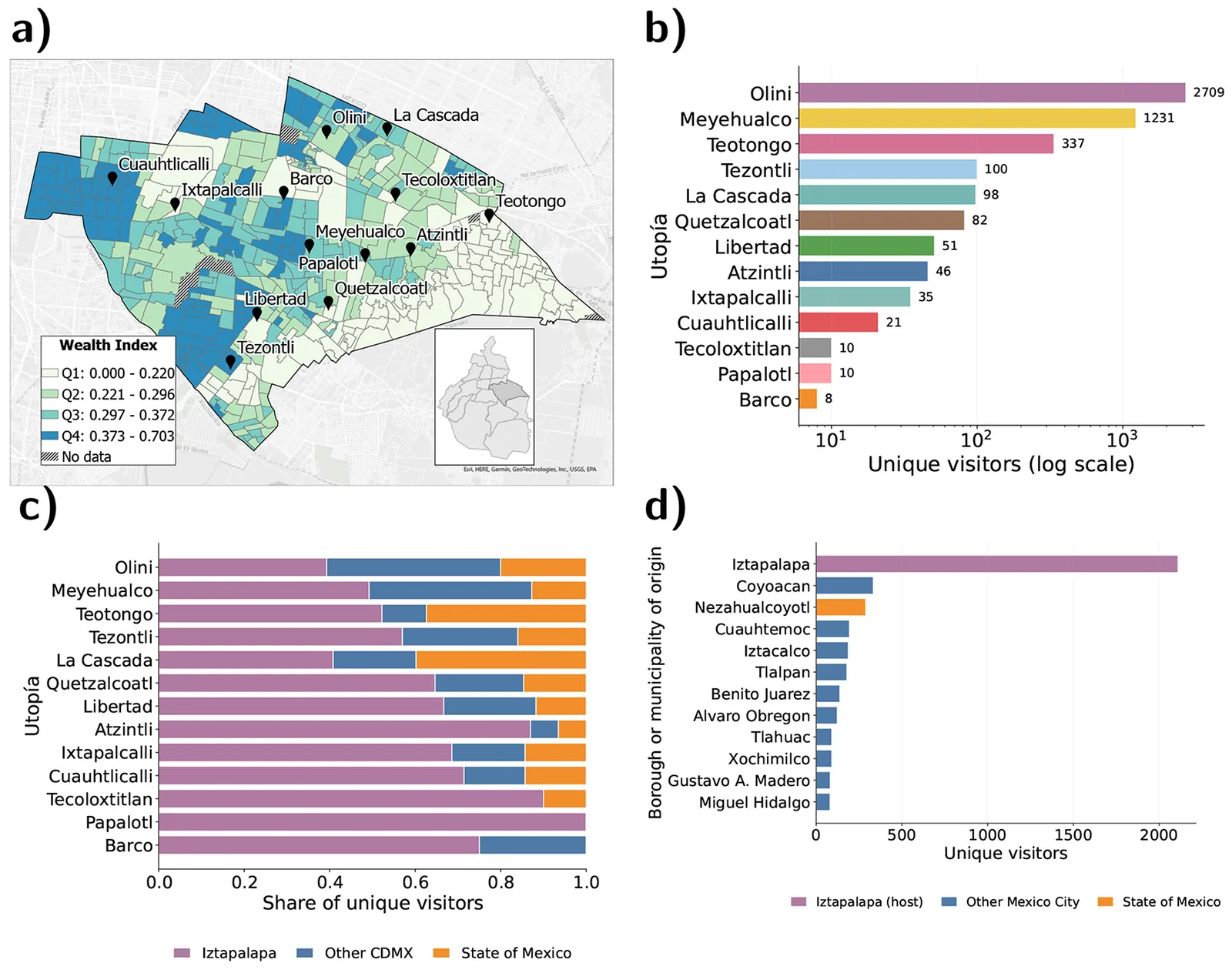
The network and who it reaches. **(a)** The 13 Utopías across Iztapalapa, over the residential wealth quartiles of the surrounding area, with an inset locating the borough within Mexico City. **(b)** Unique visitors per site on a log scale; Olini and Meyehualco are an order of magnitude above the rest. **(c)** Per-site composition of visitors by borough of origin (Iztapalapa, other Mexico City boroughs, State of Mexico); the smallest sites are almost entirely local while the two largest pull from across the metropolitan area. **(d)** The twelve largest origin boroughs across the network; Iztapalapa dominates, followed by Coyoacán, Nezahualcóyotl, Cuauhtémoc, and Iztacalco.

The spatial organization of visitor origins closely mirrors this hierarchical division (Figures 1c, d). In the aggregate, the network draws a slight majority of its visitors, approximately fifty-five percent, from outside the host borough, and roughly one in five from the neighboring municipalities of the State of Mexico. This metropolitan-scale catchment is concentrated almost exclusively in the two largest hubs, where Olini and Meyehualco draw only forty and forty-eight percent of their visitors from within Iztapalapa itself. In contrast, the smaller, peripheral neighborhood spaces remain deeply localized, with sites like Papalotl, Atzintli, and Tecoloxtitlan drawing over ninety percent of their visitors from their immediate surroundings. While cross-boundary mobility is a defining feature of the network, it is heavily polarized and concentrated at the largest, best-connected sites. These well-connected locations pull visitors from across municipal borders, and they combine proximity to metro corridors with distinctive signature attractions, whereas poorly connected spaces primarily serve to support local daily routines.

### 3.2 Temporal dynamics of visitation

The temporal evolution of network visitation is characterized by a gradual expansion from October to a peak in early February, followed by a moderate decline through March (Figure 2). This macro-level trend, rather than high-frequency weekly periodicity, constitutes the primary signal in the data. To systematically isolate these temporal scales, we apply a seasonal-trend decomposition procedure (Cleveland et al., 1990) to partition the daily visitor counts into long-term trend, weekly seasonal, and residual components. The decomposed trend component accounts for the vast majority of the observed variance, rising from an average of 20 unique daily visitors in early October to a smoothed peak of approximately sixty in early February before gradually easing.

**Figure 2:**
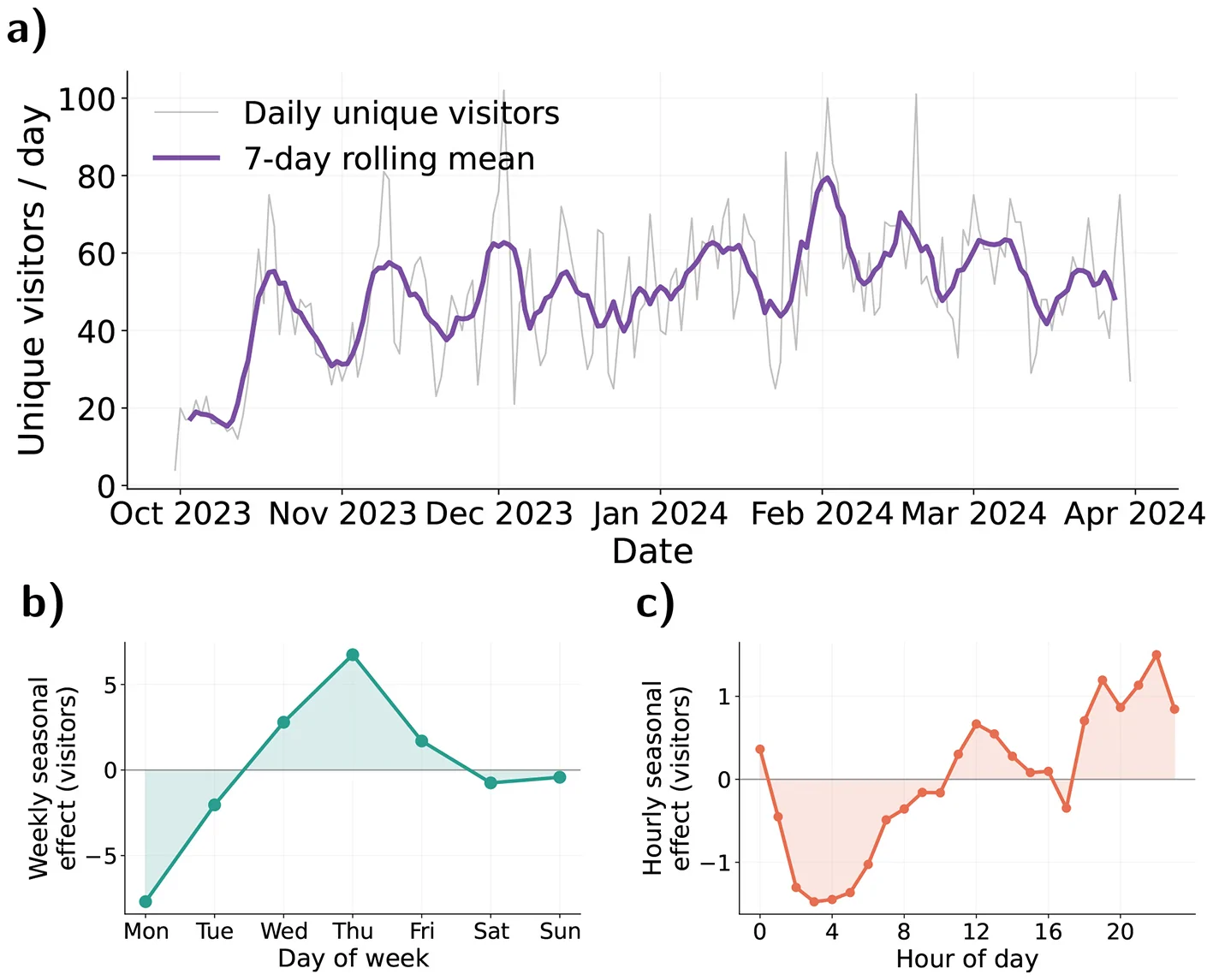
Visit dynamics and rhythm. **(a)** Daily unique visitors across the network with a 7-day rolling mean; visits increase from roughly seventeen per day in October to above seventy in early February and then ease. **(b)** The weekly seasonal component of the decomposition, small in amplitude relative to the trend. **(c)** The seasonal component by hour of day, which builds through the afternoon and into the evening. The trend component and a view by weekday and hour are shown in the Supplementary material.

In contrast, the weekly cyclical component remains relatively weak, with less than one unique visitor per day separating average weekend and weekday volumes (Figure 2b). This suggests that the Utopías are used consistently throughout the week rather than primarily as weekend destinations, with the long-term seasonal trend dominating weekly fluctuations. At the diurnal scale, however, visitation patterns reveal a well-defined progression, with activity steadily accumulating through the afternoon and peaking during evening hours (Figure 2c). Disaggregated per-site analyses, detailed in the Supplementary material, show that this overarching trend is led by the two highest-volume sites (Olini and Meyehualco). Nonetheless, the smaller neighborhood sites exhibit highly synchronized temporal trajectories, tracking the same macro-level seasonal path rather than operating on independent localized cycles.

### 3.3 Visitor wealth, travel distance, and catchment composition

Visitors are concentrated in the lower half of the metropolitan wealth distribution, although the socioeconomic composition varies substantially across sites (Table 1). Much of this between-site variation is associated with differences in geographic catchment. Median travel distances from home to site vary substantially across the network, ranging from under half a kilometer at highly localized facilities (such as Atzintli) to between seven and eight kilometers at Tecoloxtitlan and Olini. This variation in catchment distance is strongly associated with the place-level socioeconomic segregation index (*S*_*a*_).

**Table 1:** Per-Utopía summary of the metrics used in the analysis, sorted by unique visitors.

Utopía	Visitors	Med. dist. (km)	Med. wealth	*S*_*a*_	% Izta	Viol. share	Transit	Access	Comp.
Olini	2,709	7.5	0.33	0.16	40	0.029	0.94	0.80	+0.52
Meyehualco	1,231	6.7	0.34	0.18	48	0.036	0.40	0.74	+0.27
Teotongo	337	4.4	0.23	0.28	42	0.036	0.90	0.00	+0.85
Tezontli	100	1.1	0.28	0.28	64	0.040	0.76	0.17	–0.09
La Cascada	98	5.3	0.32	0.38	41	0.037	0.35	0.49	–0.29
Quetzalcoatl	82	1.7	0.22	0.20	69	0.046	0.31	0.31	–0.13
Libertad	51	5.0	0.36	0.22	68	0.032	0.23	0.54	+0.09
Atzintli	46	0.4	0.24	0.46	94	0.047	0.41	0.24	–0.72
Ixtapalcalli	35	2.9	0.28	0.51	74	0.035	0.66	0.97	+0.42
Cuauhtlicalli	21	5.0	0.34	0.21	71	0.022	0.53	1.00	+0.08
Papalotl	10	1.5	0.27	0.40	100	0.044	0.32	0.44	–0.51
Tecoloxtitlan	10	7.7	0.27	0.40	93	0.050	0.03	0.49	–0.74
Barco	8	5.0	0.32	0.33	59	0.027	0.19	0.96	+0.25

Wealth is the metropolitan wealth index (higher is wealthier); *S*_*a*_ is the place-level segregation index of Equation 5; % Izta is the share of visitors whose home is inside Iztapalapa; violent share is the fraction of within-buffer incidents in violent categories; transit and access are the transit and distance-weighted accessibility scores, each rescaled to run from zero to one; the composite is the equal-weight z-scored mean.

Figure 3a shows the relationship between *S*_*a*_ and mean catchment distance, together with the least-squares regression line and its bootstrap 95% confidence interval. We observe a statistically significant negative association (*r* = −0.60, *p* = 0.030), on a sample of thirteen sites. The same pattern is further reflected in the per-site quartile composition (Figures 3b-d). Sites with the most expansive geographic catchments, such as Olini and Meyehualco, attract a more balanced distribution across the four metropolitan wealth quartiles, thereby registering the lowest levels of *S*_*a*_. In contrast, highly localized neighborhood sites show socioeconomic profiles close to those of their immediate residential surroundings. This association suggests that sites with broader geographic catchments tend to attract a more socioeconomically diverse set of visitors. As throughout the paper, this relationship is interpreted as associational rather than causal.

**Figure 3:**
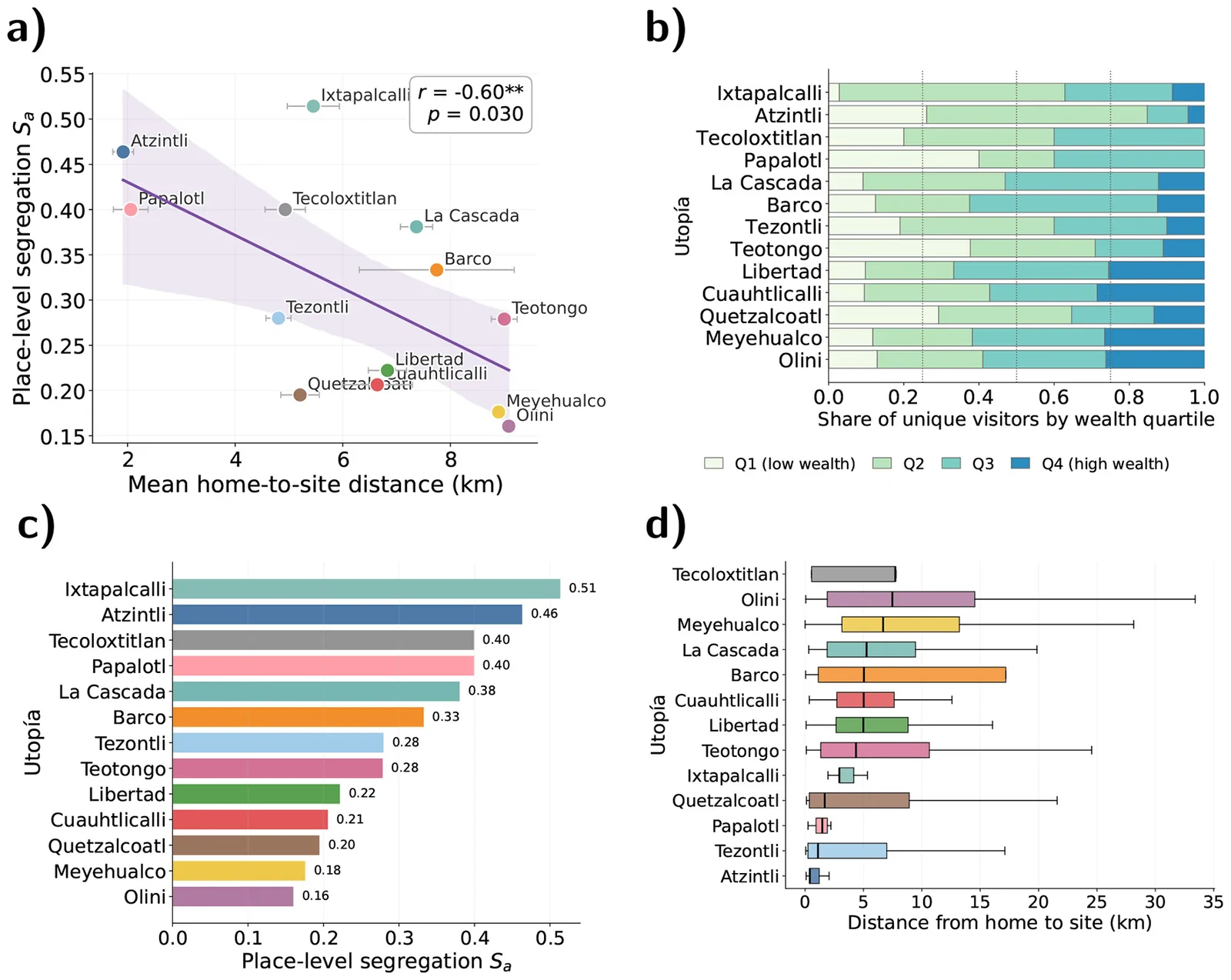
Wealth mix, distance and segregation. **(a)** Place-level wealth segregation *S*_*a*_ (Moro et al., 2021) against mean distance from home to site (horizontal bars are the standard error of the mean distance); the negative slope (*r* = −0.60, *p* = 0.030) is consistent with sites that draw from farther attracting a more even mix of wealth quartiles. **(b)** Per-site composition of unique visitors by metropolitan wealth quartile (Q1 low wealth to Q4 high wealth), ordered by *S*_*a*_; dotted lines mark the even-mix shares. **(c)** *S*_*a*_ per site. **(d)** Distribution of the distance from home for each site, ordered by median.

### 3.4 Service distinctiveness and travel distance

We examine the three site profiles in turn, starting with what each Utopía offers. The richness of the offer has little association with visitor volume. Across the broad attributes of the catalog (Figure 4a), neither the facility count, the number of landmark attractions, nor the distinctiveness index is correlated with unique visitors, with the distinctiveness index against visitors giving *r* = 0.05. Instead, the offer is associated with how far people travel to visit a site, particularly with the weekend distance premium. Rarer, more capital-intensive, and more distinctive portfolios are associated with a larger premium (site-level distinctiveness against the premium, *r* = 0.68, *p* = 0.015; Figure 4c).

**Figure 4:**
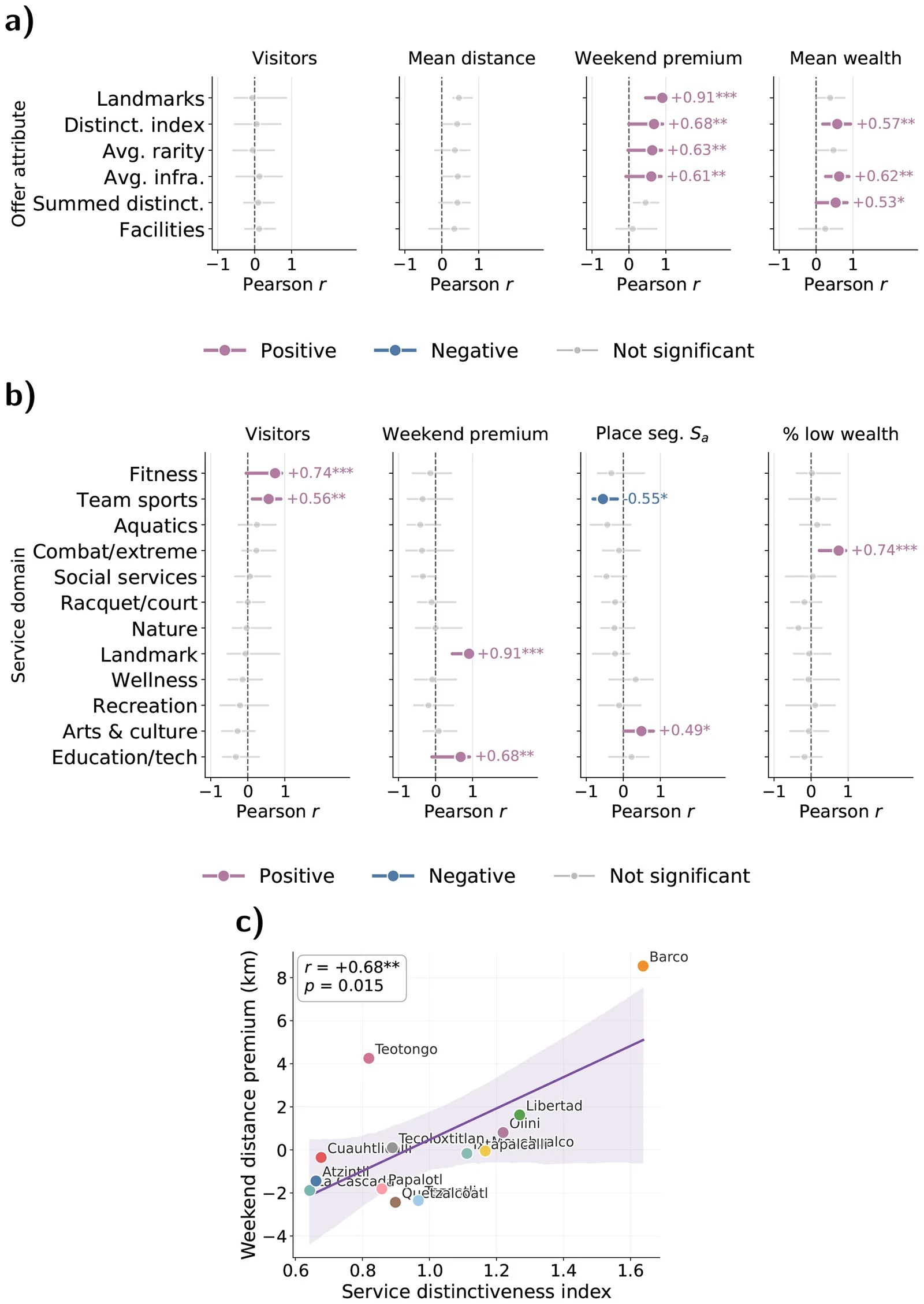
The service offer and how people travel for it. **(a)** Pearson correlations, with bootstrap 95% confidence intervals, between six summaries of the offer and four visit outcomes; the weekend distance premium and mean visitor wealth respond, the visitor count does not. **(b)** The facility count in each of twelve service domains against four outcomes. **(c)** Site-level distinctiveness against the weekend distance premium (*r* = 0.68, *p* = 0.015). Intervals are colored where the permutation *p* is below 0.10 and gray otherwise; ^***^p < 0.01, ^**^p < 0.05, ^*^p < 0.10.

Resolving the catalog into twelve service domains (Figure 4b) reveals a clearer pattern and separates two kinds of offer. Everyday, repeat-use facilities go with higher visitor volumes. Sites with more fitness and more team-sports facilities have more unique visitors (*r* = 0.74, *p* = 0.004, and *r* = 0.56, *p* = 0.044), and fitness provision is associated with lower recurrence (*r* = −0.60, *p* = 0.029), consistent with a high share of one-off gym users. Team sports show the opposite pattern, falling with both the weekend share (*r* = −0.53, *p* = 0.064) and place-level segregation (*r* = −0.55, *p* = 0.051), a profile consistent with neighborhood league use that is local and weekday-leaning but socially even, while aquatics lean toward weekend use without reaching significance (*r* = 0.34).

The signature end of the offer shows a different pattern. Landmark attractions, and to a lesser extent the education and technology domain and rare offerings, are not associated with visitor volume but are strongly associated with the weekend distance premium (landmark domain against premium, *r* = 0.91, *p* < 0.001; education and technology, *r* = 0.68, *p* = 0.011). In other words, signature attractions track how far visitors travel rather than how many arrive, particularly on weekends. The finer analysis at the level of each individual activity, including the effect of adding or removing a given service, is reported in the Supplementary material.

### 3.5 Transit access and visitor volume

While the service offer is associated with how far visitors travel, public transport infrastructure is the strongest correlate of aggregate visitor volumes. The composite transit accessibility score correlates strongly with log-scaled unique visitors (*r* = 0.64, *p* = 0.019; Figure 5b), representing the most prominent univariate association with volume within our empirical sample. This pattern indicates that visitation is more closely associated with the physical supply and capacity of transit services than with geographic proximity alone. A conventional distance-weighted accessibility index, the classic formulation of potential accessibility, exhibits no significant relationship with empirical visitation (*r* = −0.12, *p* = 0.684). A site's actual reach lines up more closely with network connectivity and transit supply than with its central positioning within the urban geography.

**Figure 5:**
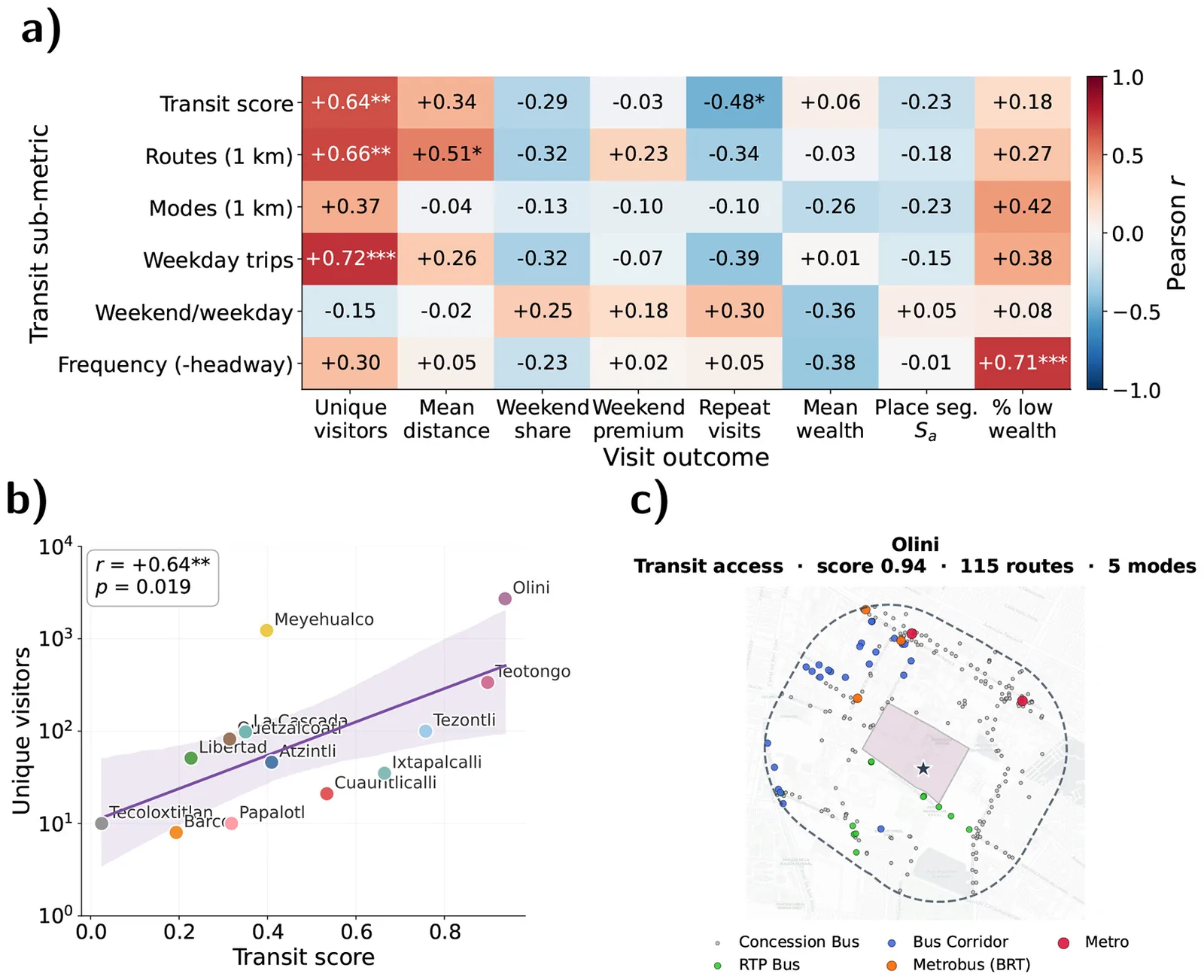
Transit access and visit volume. **(a)** Pearson correlations between the transit sub-metrics within the one-kilometer buffer (the transit score, route count, mode count, weekday trips, the ratio of weekend to weekday trips, and service frequency taken as the negative of peak headway) and eight visit outcomes; routes and weekday trips carry the visitor signal. **(b)** The transit score versus unique visitors on a log scale (*r* = 0.64, *p* = 0.019). **(c)** The transit environment of a well-connected site: GTFS stops within Olini's one-kilometer perimeter buffer, colored by mode, over which the sub-metrics in (a) are computed. The dashed line is the buffer, the shaded polygon the site, and the star its centroid. Cells marked ^***^p < 0.01, ^**^p < 0.05, ^*^p < 0.10.

Decomposing the composite transit score into its constituent metrics (Figure 5a) isolates the components that carry this association: the total number of unique transit routes and the volume of weekday trips within the one-kilometer buffer carry the majority of the predictive signal. Conversely, temporal service characteristics, such as headways or the ratio of weekend to weekday service, show negligible associations, while the presence of heavy rail (metro) and Bus Rapid Transit (BRT) is relevant mainly for the network's two largest sites. Figure 5c illustrates this transit environment at Olini, the most highly connected node in the network, where metro, BRT, and high-density local feeder routes intersect within its immediate catchment buffer.

Transit connectivity shows little association with the socioeconomic composition of visitors. Transport infrastructure is therefore associated primarily with the overall scale of visitation, the quantity of visits, rather than with the socioeconomic sorting of visitors. Well-connected sites show no systematic tilt toward wealthier visitor catchments or more integrated visitor profiles; rather, they are associated with a larger overall volume of users.

### 3.6 Surrounding violence and visitor composition

The volume of crime around a site is not associated with how many people visit it. The total volume of crime within a site's buffer, whether measured as a raw count or spatial density, shows no association with its total visitation (*r* = −0.01). This runs against the expectation, common in the literature on park use, that perceived danger suppresses visitation (Foster et al., 2014). Some property-crime categories, such as delivery-worker, pedestrian, and taxi robbery, exhibit positive associations with visitor volumes (Supplementary material). This pattern is consistent with a common spatial agglomeration effect, wherein both pedestrian footfall and opportunities for opportunistic crime concentrate within highly accessible, central urban nodes. Rather than tracking visitor volume, surrounding crime is more strongly associated with the demographic composition and spatial reach of the catchment area.

The share of violent incidents within a site's buffer is tightly and negatively associated with both the mean wealth of its visitors (*r* = −0.75, *p* = 0.004; Figure 6a) and their travel distances from home to site (*r* = −0.64, *p* = 0.019; Figure 6b). Consequently, facilities in areas with higher rates of violent crime are associated with a structurally distinct visitor demographic, drawing almost exclusively from poorer, highly localized populations. Decomposing this violent share into specific crime categories (Figure 6c) reveals that the negative association with visitor wealth is led by the prevalence of rape (*r* = −0.74, *p* = 0.006) and violent business robbery (*r* = −0.71, *p* = 0.006). The violent share also exhibits a moderate but non-significant positive correlation with place-level segregation (*r* = 0.41, *p* = 0.164; Figure 6d).

**Figure 6:**
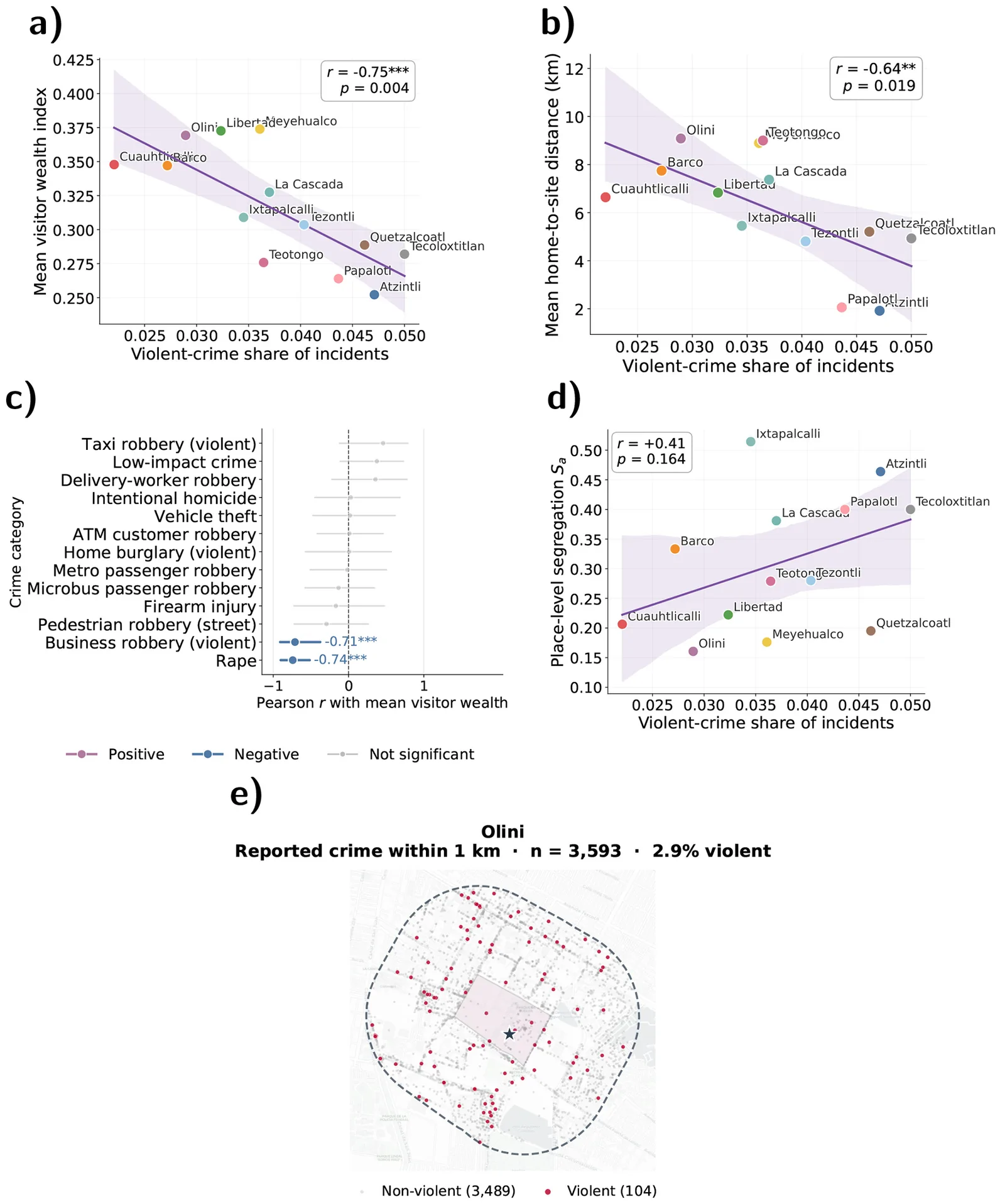
Surrounding violence and who is reached. **(a)** The violent share of crime within the one-kilometer buffer against the mean wealth of a site's visitors (*r* = −0.75, *p* = 0.004). **(b)** Violent share against the mean distance from home to site (*r* = −0.64, *p* = 0.019): more violent surroundings go with shorter, more local catchments. **(c)** Per-category correlation between the within-buffer share of each crime category and mean visitor wealth; rape and violent business robbery lead the negative associations. **(d)** Violent share against place-level segregation *S*_*a*_ (*r* = 0.41). **(e)** Reported crime within Olini's one-kilometer buffer over 2023 and 2024, with violent incidents highlighted (104 of 3,593; 2.9% violent). The raw-volume crime measures, which do not track visits, are shown in the Supplementary material.

We do not interpret these associations as crime directly determining individual destination choices or wealth levels. Instead, they coincide with the overlapping geographies of violence and residential poverty within Iztapalapa. One mechanism consistent with this pattern is that residents of high-violence neighborhoods visit their nearest Utopía while wealthier residents, fewer of whom live in those neighborhoods, travel to safer and better-connected sites. We observe where visitors live and which site they reach, not why they chose it, so we put this forward as a hypothesis rather than a result. What the data do show is that the composition of local crime, rather than its absolute volume, is the aspect that tracks the demographic a facility serves. This spatial pattern is illustrated in Figure 6e for the case of Olini, where violent crimes constitute a small, spatially scattered minority within a high-volume crime buffer.

### 3.7 The three dimensions combined

Figure 7a sets the three dimensions, and their equal-weight composite, against the visit outcomes. The three barely overlap, with transport access tracking visitor volume, the service offer the weekend distance premium, and the crime profile visitor wealth. The composite inherits the volume signal, correlating with unique visitors (*r* = 0.54, *p* = 0.056; Figure 7b), almost entirely through its transit component.

**Figure 7:**
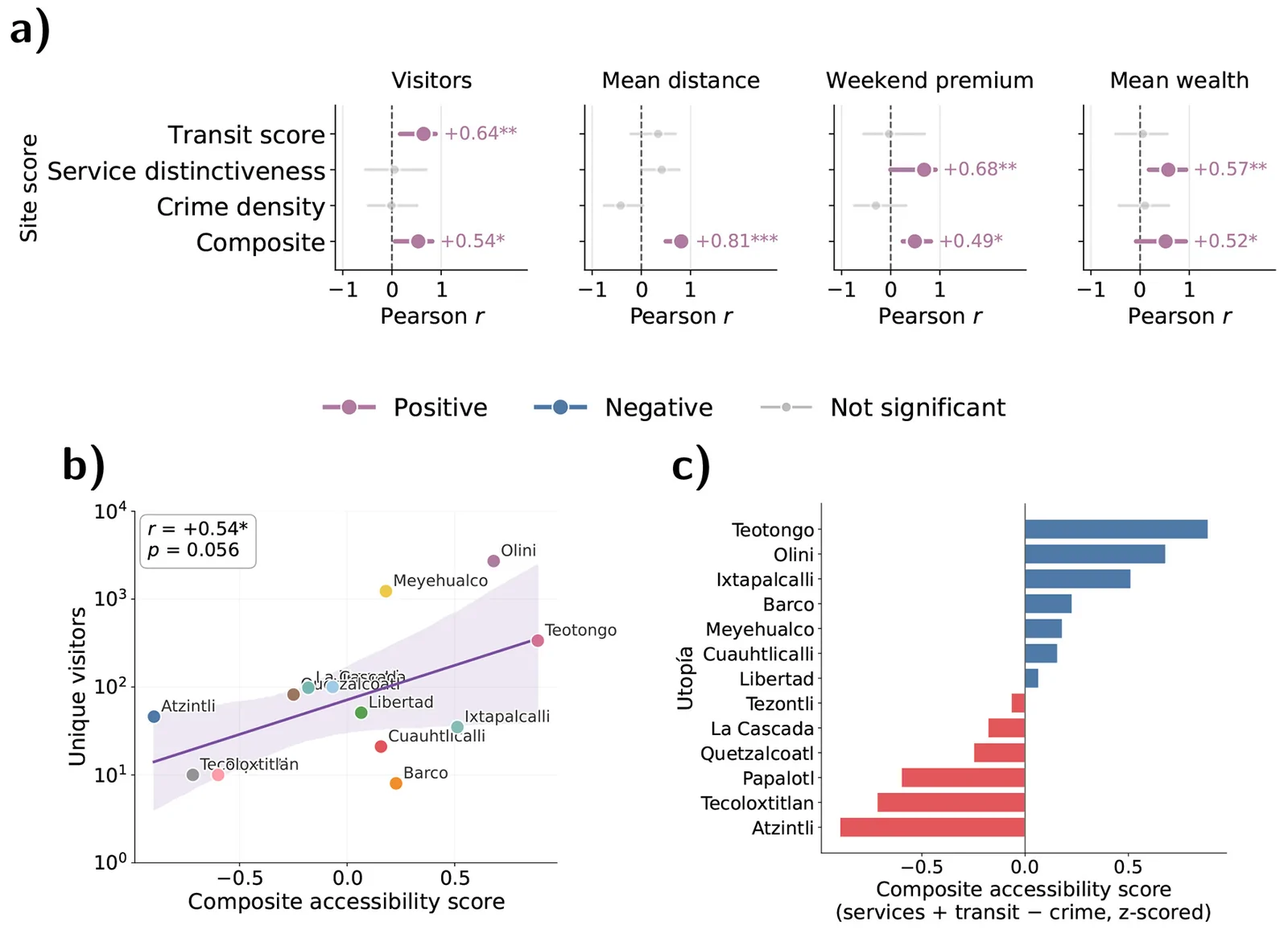
The combined accessibility index. **(a)** Pearson correlations, with bootstrap 95% confidence intervals, between the four site scores (transit, service distinctiveness, crime density, and the equal-weight composite) and four visit outcomes; each dimension tracks a different facet of reach. **(b)** Composite score versus unique visitors on a log scale (*r* = 0.54, *p* = 0.056). **(c)** Composite accessibility score per site (services plus transit minus crime, equal weights, z-scored). Intervals are colored where the permutation *p* is below 0.10 and gray otherwise; ^***^p < 0.01, ^**^p < 0.05, ^*^p < 0.10.

Transit holds this association on its own. Controlling for services and crime leaves it unchanged (partial *r* = 0.65, *p* = 0.016), and in a standardized regression of log visits on the three axes it takes a coefficient of 0.65 against 0.15 for the service offer and −0.02 for crime (*R*^2^ = 0.44). The composite ranking itself (Figure 7c) puts Teotongo and Olini at the top, the two best-connected sites in the network, and Tecoloxtitlan, Atzintli and Papalotl at the bottom. Against mean travel distance the composite tracks more closely than any of its three parts on its own (*r* = 0.81, *p* = 0.001, against 0.34 for transit, 0.41 for the service offer and −0.42 for crime density). The same relationship computed on median rather than mean distance is weaker and not significant (*r* = 0.36, *p* = 0.216). This behavior is consistent with the hypothesis that each independent axis is associated with a distinct facet of spatial reach, rather than acting on a single, shared dimension of performance. Extended analyses, including site-level profiles of the three axes, detailed transit sub-metrics, and the composite index under alternative weighting schemes, are provided in the Supplementary material.

### 3.8 A typology of site access

We cross the two levers that are most directly under local government control, transit accessibility and the distinctiveness of the service offer, to partition the network into four site types (Figure 8). We split each axis at its median across the thirteen sites, 0.40 on the rescaled transit score and 0.90 on the distinctiveness index, shown as the dashed lines in the figure. Types are therefore defined relative to the rest of the network, and each holds at least three sites. *Flagship destinations* are above the median on both axes, combining good transit connectivity with amenities found nowhere else in the network: Olini, Meyehualco, Tezontli, and Ixtapalcalli. *Connected neighborhood hubs* are well served by transit but offer a standard recreation package: Teotongo, Atzintli, and Cuauhtlicalli. *Under-connected attractions* offer distinctive services in a sparse transit environment: Libertad, Barco, and Quetzalcoatl. *Neighborhood spaces* have neither extensive transit infrastructure nor specialized services, serving primarily as localized assets for the daily routines of their immediate surroundings: La Cascada, Papalotl, and Tecoloxtitlan.

**Figure 8:**
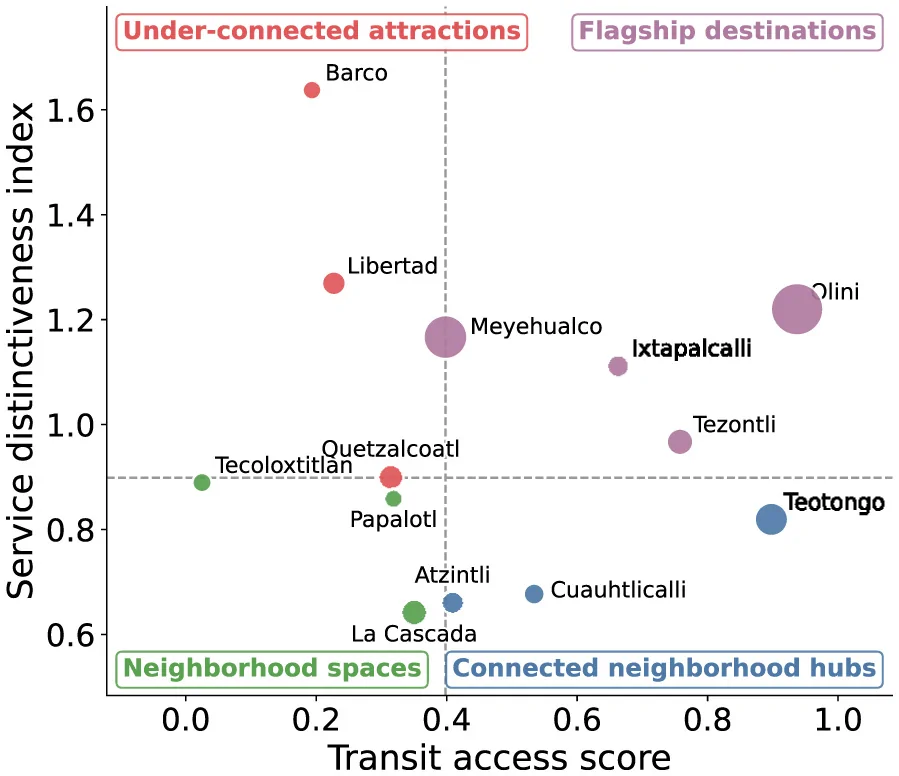
Access typology of the network, from transit access against the service distinctiveness index, with marker size proportional to unique visitors and color denoting the four types: flagship destinations (wide transit and a distinctive offer), connected neighborhood hubs (wide transit, standard offer), under-connected attractions (distinctive offer, thin transit), and neighborhood spaces (neither). The dashed lines are the network medians that separate the four types, 0.40 on the rescaled transit score and 0.90 on the distinctiveness index.

The typology lets us compare sites at differing levels of each dimension, which is the comparison a government faces when deciding where to build and what to put inside.

## 4 Discussion

The Utopías draw visitors from across the metropolitan area, and most of those visitors come from the lower half of the wealth distribution. Their reach varies across sites, and three site characteristics are associated with much of that variation: transit accessibility, the service offer and surrounding crime, which we take in turn.

Transport accessibility is the primary correlate of absolute visitor volume. In an environment where local trips rely on colectivos and the two largest sites sit on metro and BRT corridors, local transit supply is strongly associated with visitor volume. Transit connectivity accounts for nearly all of the combined index's association with volume when controlling for other factors, aligning with established literature identifying transit barriers for non-driving populations (Wolch et al., 2014; Boone et al., 2009; Sister et al., 2010; Bocarejo and Oviedo, 2012; Vecchio et al., 2020). This also offers a highly cost-effective planning tool, since GTFS data are public and spatial buffers are straightforward, so transit screening can occur before capital commitment.

The service offer is associated with travel distance rather than visitation volume, appearing as a weekend distance premium. Sites with highly specialized signature attractions (e.g., planetariums or dinosaur parks) record longer visitor travel distances on weekends, whereas standard recreational facilities support localized daily routines. Landmark amenities are the strongest correlate of this travel premium. Our small sample size (*n* = 13) warrants caution, as site scale and transit access are somewhat confounded with distinctiveness, but one implication for policy follows. For municipal programs weighing signature infrastructure, the return appears to lie in extending a site's metropolitan catchment on weekends rather than in raising daily visitor volume.

Surrounding crime shows two distinct patterns of association. Its absolute volume is unrelated to visitation, while the violent share carries a strong, negative association with visitor wealth and travel distance. Absolute crime tends to concentrate within highly accessible, high-footfall nodes. Conversely, the violent share correlates with who visits rather than how many, reflecting the overlapping geographies of violence and residential poverty in Iztapalapa. A plausible reading, which our design cannot test, is that poorer residents use local, high-violence Utopías while wealthier residents visit safer, better-connected sites. A public space's visitor composition thus reflects its immediate residential geography, with the composition of local crime, rather than its volume, marking the difference. This aligns with Latin American literature showing that localized street-level safety, rather than aggregate crime counts, is what matters for public space access among vulnerable groups, particularly women (Loukaitou-Sideris, 1995; Carmona et al., 2010; Foster et al., 2014; Sánchez De Madariaga and Arvizu Machado, 2025).

Keeping the three dimensions separate is what makes the individual mechanisms visible, but the combined score is informative beyond its components for one outcome. The composite tracks the mean distance visitors travel (*r* = 0.81, *p* = 0.001) more closely than the transit score (0.34), the service offer (0.41), or crime density (−0.42) taken separately, none of which is significant on its own. Sites that draw visitors from across the metropolitan area therefore appear to score well on several dimensions at once, a pattern that becomes visible only when the dimensions are considered together. We interpret this cautiously, since the relationship is specific to mean rather than median distance. Together, these dimensions resolve equity questions that traditional administrative records cannot. While the Utopías successfully serve residents across the socioeconomic spectrum, the network exhibits a steep hierarchy that mirrors existing socio-spatial inequalities. The two sites capturing the highest volumes, widest catchments, and most diverse visitor pools are also the best served by rapid transit. Conversely, localized sites in violent surroundings serve the most vulnerable and spatially constrained populations. These findings suggest that the network's greatest equity challenges lie where access remains local, visitor populations are most vulnerable, and conventional monitoring provides the least information about actual use.

Observing the sites for 6 months also shows something a one-time survey would miss. Visits at most sites grew steadily from October to February. The weekly cycle is flat, so people use the sites during the week and not only on weekends. The same data could be used to follow new sites during their first months and see which ones are picking up and which are not. This would help the borough decide where to adjust programming or transit, and which kinds of sites to build next.

### 4.1 Implications for the expansion

The three dimensions have direct implications for how a program like this can evaluate candidate sites as it expands, whether to other boroughs of Mexico City or to other cities with the same deficits. Among the three, transit access is the easiest to act on and the cheapest to assess. It can be screened with public GTFS data before a single facility is built, and it is the dimension most strongly associated with visitor volume. A transit pre-screen of candidate locations is therefore a high-return diagnostic that requires no new data collection or fieldwork.

The second implication is a caution about how to interpret the wealth mix a site achieves. A facility placed in a homogeneously low-income area will tend to draw a narrow wealth distribution regardless of its design, while the same facility in a more mixed area would record lower place-level segregation simply because of its surroundings. Segregation indices reflect the residential geography around a site as much as anything the program controls, and they should be read against the local baseline rather than treated as a verdict on the facility. Expecting a site in a poor periphery to reach the wealth mix of a centrally located one sets a standard the evidence does not support.

The third implication concerns the return on signature infrastructure. A planetarium, a wave pool, or a dinosaur park is not associated with substantially higher daily visitor volume, but sites that have one draw visitors from across the metropolitan area on weekends, extending the catchment well beyond the immediate neighborhood. Whether that reach justifies the capital cost depends on the goal. If the goal is to serve a wide and diverse population, signature attractions deliver something real. If it is to maximize daily use by the residents around a site, the same money spent on transit connectivity or everyday recreation infrastructure would likely do more.

### 4.2 Limitations

Our analysis shares the limitations common to studies built on LBS data. Smartphone and app penetration are lower among lower-income populations, exactly the population the Utopías are meant to serve, so our raw counts under-represent the most vulnerable users (Cabrera and Rowe, 2026; Coston et al., 2021; Wesolowski et al., 2013); the home-detection and residential-wealth steps help mitigate this limitation at the residential scale but cannot recover users who never appear in the data. We also observe a single GTFS snapshot and a single six-month window, so we cannot speak to how reach responds to changes in service or investment, though repeated windows are the obvious next step, and our pipeline is readily applicable for them. The crime registry covers only reported incidents, and under-reporting is itself uneven across categories (INEGI, 2024); reported crime is in any case a coarse proxy for the street-level perception of safety that the literature identifies as the operative filter. The LBS panel is not disaggregated by sex, so we cannot test whether women's visitation is more sensitive than men's to these safety gradients, which leaves the program's care-oriented goals a question for field-based research. The segregation index *S*_*a*_ is computed from unique visitors and is noisy for the smallest sites (Barco, Papalotl, Tecoloxtitlan, all in the single or low double digits), so the network rankings should be interpreted with appropriate caution. Finally, with thirteen sites and one window, every relationship reported here is a correlation. Separating, for example, the effect of transit from the borough's choice to place its largest sites where transit already existed would need either an instrument or future analyses as the program expands to additional boroughs.

## 5 Conclusion

The Utopías of Iztapalapa bring publicly funded recreation, culture, science and care to a borough that has long lacked all of them, and mobility data let us see who reaches them at low cost. Over six months the network attracted 4,704 unique visitors (the per-site counts in Table 1 sum to slightly more, since a few visitors appear at more than one site). The distribution is steep: two large, well-connected sites account for most of the traffic and draw a slight majority of their visitors from across the metropolitan area, while the smallest sites serve a stable and almost entirely local group. Transport accessibility is the factor most strongly associated with visitor volume. The distinctiveness of a site's amenities is strongly associated with how far people travel on weekends, and the share of violent crime in the surrounding area is linked to the socioeconomic profile of visitors more than to attendance. The socioeconomic composition of a site's users also becomes more balanced as its catchment grows. The program is reaching people, but the sites with the least reach are in the parts of the borough where deficits are deepest. Infrastructure alone is unlikely to close that gap without improvements in transport and safety around the sites. None of these relationships is a causal estimate. Together they give a borough a baseline against which to measure new sites, and a short list of levers, transport access first among them, that any city can screen from open data before a single facility is built.

## Data Availability

The datasets presented in this study can be found in online repositories. The names of the repository/repositories and accession number(s) can be found in the article/supplementary material.

## Footnotes

1
https://datos.cdmx.gob.mx/dataset/wifi-gratuito-en-utopias-de-la-cdmx

2
https://utopias.mx/
