# Supplementary material for: Evaluating the reach and equity of public spaces in Mexico City using mobility data: the case of the Utopías in Iztapalapa
Source: Front Big Data. 2026 Sep 23;9:1942458. doi: 10.3389/fdata.2026.1942458 (PMC13642205; doi:10.3389/fdata.2026.1942458)
Supplement: Supplementary file 1 [file Supplementary_file_1.pdf]

Supplementary Information

S1 Statistical inference

All correlations are Pearson correlations on the thirteen-site cross-section, with two-sided permutation p-values (up to 10,000 permutations) and bootstrap 95% confidence intervals (up to 5,000 resamples); for the heavily skewed visit counts we correlate on the base-ten logarithm. Partial correlations are computed on linear residuals, controlling for the mean of the remaining two axes. The standardized regression of log visit volume on the three axes gives coefficients +0.65 (transit), +0.15 (services) and −0.02 (crime), with $R^2 = 0.44$.

S2 Summary of correlations

Table S1 collects every association reported in the main text in one place, pairing each per-site predictor with each visit outcome so that the individual results can be read against the full set rather than one at a time.

Table S1 Pearson correlations between per-utopia predictors and visit outcomes (** $p < 0.05$, * $p < 0.10$); the S_a versus mean-distance row reports a least-squares fit (R^2).

Predictor	Outcome	r	p	sig.
Transit score	Unique visitors (log)	+0.64	0.019	**
Transit score	Median distance	−0.09	0.766	–
Transit score	% low wealth	+0.18	0.554	–
Transit score	Place seg. S_a	−0.23	0.452	–
Distinctiveness index	Unique visitors (log)	+0.05	0.858	–
Distinctiveness index	Recurrence	−0.25	0.424	–
Distinctiveness index	Weekend share	+0.17	0.560	–
Distinctiveness index	Mean distance	+0.41	0.159	–
Distinctiveness index	Weekend dist. premium	+0.68	0.011	**
Landmark items	Weekend dist. premium	+0.91	< 0.001	**
Crime density	Unique visitors (log)	−0.01	0.974	–
Crime density	Mean wealth	+0.10	0.748	–
Violent share	Unique visitors (log)	−0.17	0.579	–
Violent share	Mean wealth	−0.75	0.004	**
Composite score	Unique visitors (log)	+0.54	0.056	*
Composite score	Mean wealth	+0.49	0.094	*
Composite score	Median distance	+0.30	0.322	–
Composite score	% low wealth	−0.03	0.913	–
Accessibility (gravity)	Unique visitors (log)	−0.12	0.684	–
S_a (OLS fit)	Mean distance	$r = -0.60$, $R^2 = 0.37$, $p = 0.029$		**

S3 Visitor wealth and segregation

The wealth quartile cutpoints used in the Moro index S_a [1] are computed once on the full Mexico City wealth map and then applied uniformly to all sites, falling at the 25th, 50th and 75th percentiles of the metropolitan distribution. We use these fixed

cutpoints rather than per-site cutpoints because the index is meant to compare each site against a common metropolitan baseline and not against its own catchment. The index $S_a = \frac{2}{3} \sum_q |\tau_{aq} - 0.25|$ is bounded between zero (an even mix across the four quartiles) and one (all visitors in a single quartile).

S4 The service offer

Equation (3) weights each category a site offers by two quantities, its rarity ρ_c and its infrastructure intensity ι_c. The intensity is a three-point score separating standard services, specialized facilities and capital-intensive signature attractions. The site score is the mean of those weighted products over the categories the site offers. Three of these choices could reasonably have been made otherwise: whether the two terms multiply or enter on their own, how rarity is scaled, and whether the site score is a mean, a sum or a maximum. We rebuilt the index under eight variants and correlated each with the weekend distance premium (Table S2).

The association is positive under all eight, from $r = +0.38$ to $r = +0.70$, and the variants correlate with the main-text index between +0.73 and +1.00, so the result does not depend on the formula. Aggregation does affect stability. The mean used in the main text has the widest leave-one-out range, [+0.30, +0.82], because a site with a short catalog of rare offerings receives a high mean. Barco is the case in point, holding the largest weekend premium in the network on eight matched visitors, and dropping it moves the correlation to +0.30. Aggregations less sensitive to catalog length are steadier, and averaging the three most distinctive categories under an inverse-document-frequency rarity is the steadiest ($r = +0.58$, leave-one-out [+0.43, +0.65]). We keep the main-text formulation, which sits mid-range and was specified in advance.

Among the twelve service domains, the count of landmark attractions carries the relationship more robustly than the aggregate index, at $r = +0.91$ ($p < 0.001$, bootstrap 95% CI [+0.45, +0.98]), with a leave-one-out range of [+0.71, +0.94] and $r = +0.76$ ($p = 0.017$) among the nine sites with at least twenty-five matched visitors. Entering both predictors into a single standardized regression yields a coefficient of $\beta = +1.00$ ($p = 0.001$) for the landmark count and $\beta = -0.12$ ($p = 0.605$) for the index. We do not replace the aggregate index with the landmark count in the main text, since nine of the thirteen sites hold no landmark and the count behaves almost as a binary indicator here. The weekend premium is nonetheless associated more closely with having a signature attraction than with offering a generally distinctive mix of services.

Table S2 The service distinctiveness index against the weekend distance premium under alternative formulations. Pearson r with two-sided permutation p and a bootstrap 95% confidence interval, thirteen sites. The leave-one-out range is the smallest and largest r obtained when each site is dropped in turn, and the $n \geq 25$ column restricts to the nine sites with at least twenty-five matched visitors. The last column is the Pearson correlation between each variant and the formulation used in the main text, marked *, so values near +1 indicate a variant that orders the sites almost identically. IDF denotes an inverse-document-frequency rarity, $\log(N/n_c)$.

Formulation	r	p	95% CI	Leave-one-out	$n \geq 25$	Pearson corr.
Mean of $\rho_c \iota_c$ *	+0.68	0.015	[−0.00, +0.91]	[+0.30, +0.82]	+0.31	—
Sum	+0.45	0.119	[+0.13, +0.80]	[+0.40, +0.51]	+0.39	+0.78
Mean of top three	+0.56	0.041	[+0.19, +0.80]	[+0.41, +0.61]	+0.46	+0.92
Maximum	+0.38	0.187	[+0.09, +0.68]	[+0.32, +0.46]	+0.36	+0.73
Rarity alone	+0.63	0.020	[−0.02, +0.88]	[+0.30, +0.76]	+0.25	+0.94
Intensity alone	+0.61	0.028	[−0.08, +0.87]	[+0.21, +0.74]	+0.27	+0.96
IDF rarity, $\log(N/n_c)$	+0.70	0.013	[+0.01, +0.93]	[+0.33, +0.85]	+0.32	+1.00
IDF rarity, mean of top three	+0.58	0.029	[+0.21, +0.82]	[+0.43, +0.65]	+0.47	+0.94

Table S3 Per-utopia services profile, sorted by the distinctiveness index. The index is the rarity-weighted infrastructure-intensity score normalized by the number of facilities.

Utopía	Facilities	Landmark	Aquatics	Avg. rarity	Distinctiveness
Barco	14	7	1	0.83	1.64
Libertad	24	2	2	0.73	1.27
Olini	14	2	2	0.70	1.22
Meyehualco	18	1	2	0.68	1.17
Ixtapalcalli	22	0	1	0.71	1.11
Tezontli	14	0	3	0.65	0.97
Quetzalcoatl	22	1	2	0.66	0.90
Tecoloxtitlan	16	0	1	0.69	0.89
Papalotl	13	0	3	0.57	0.86
Teotongo	20	2	2	0.60	0.82
Cuauhtlicalli	15	0	2	0.57	0.68
Atzintli	10	0	1	0.54	0.66
La Cascada	14	0	2	0.51	0.64

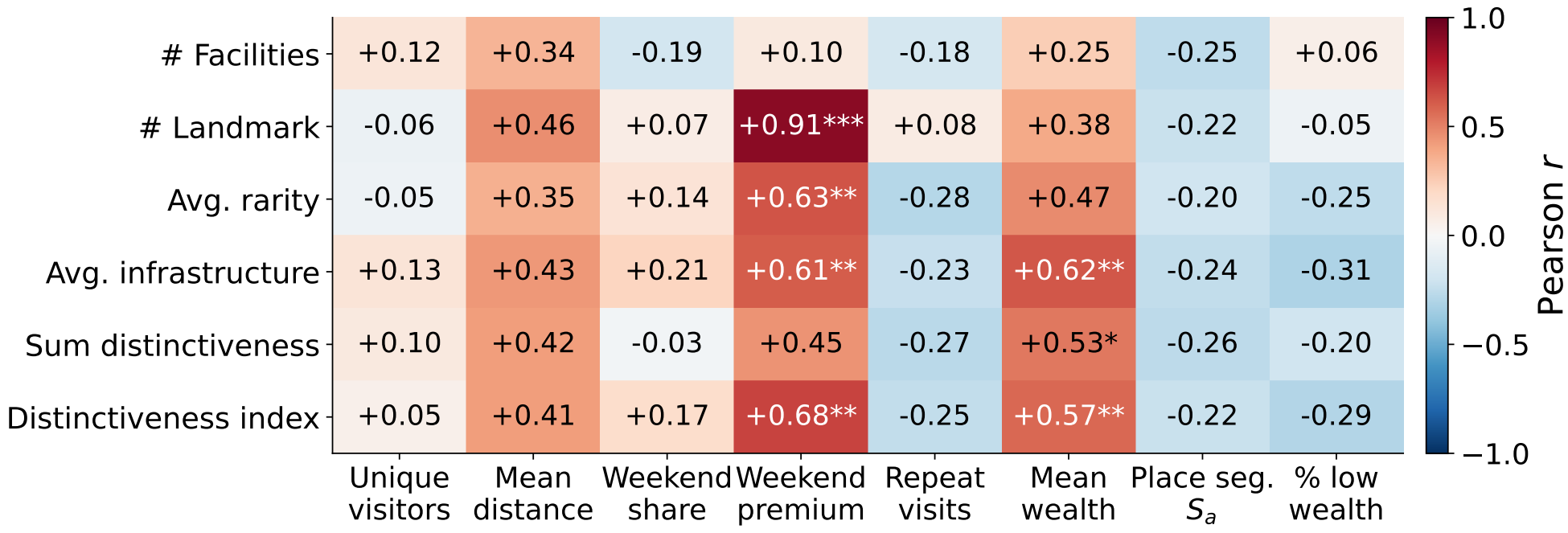

Fig. S1 Summaries of the service offer against the full set of visit outcomes. Pearson correlations between the six summaries of the offer and all eight per-site outcomes, the complete matrix behind Figure 4a of the main text, which shows the four outcomes that carry the argument. Cells marked ** $p < 0.05$, * $p < 0.10$.

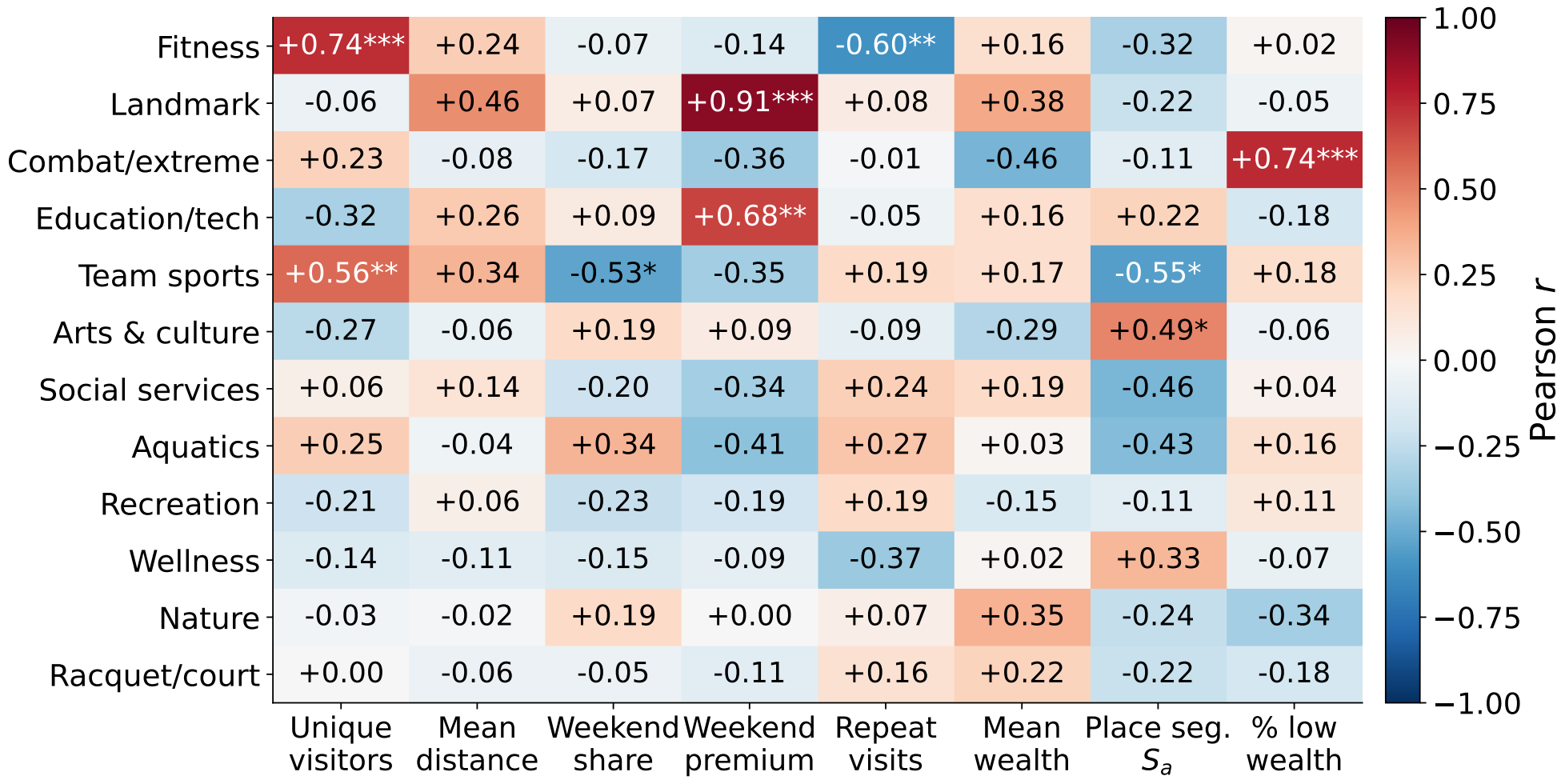

Fig. S2 Service domains against the full set of visit outcomes. Pearson correlations between the facility count in each of the twelve service domains and all eight per-site outcomes, the complete matrix behind Figure 4b of the main text. Cells marked ** $p < 0.05$, * $p < 0.10$.

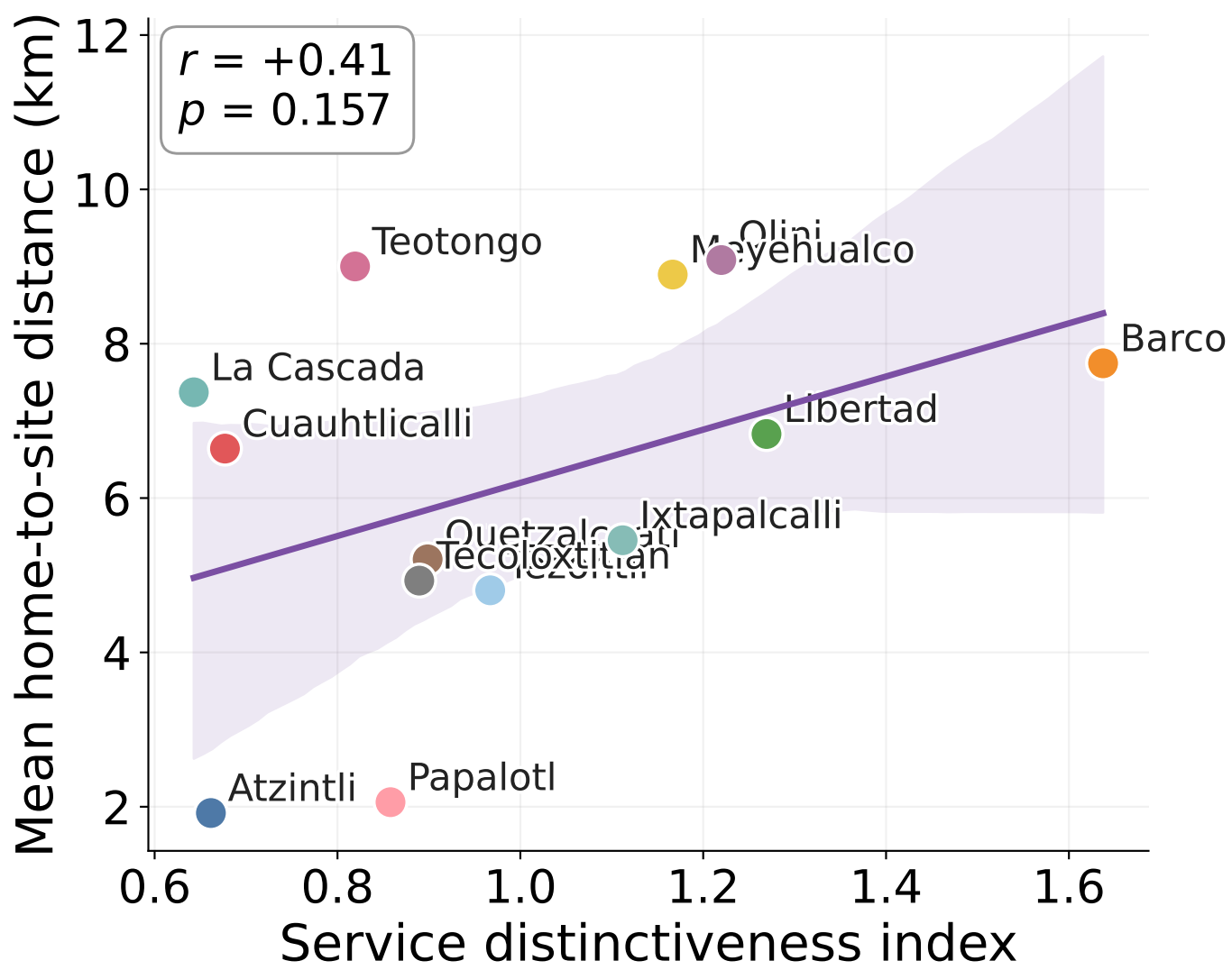

Fig. S3 Service distinctiveness against mean catchment distance ($r = 0.41$, $p = 0.16$).

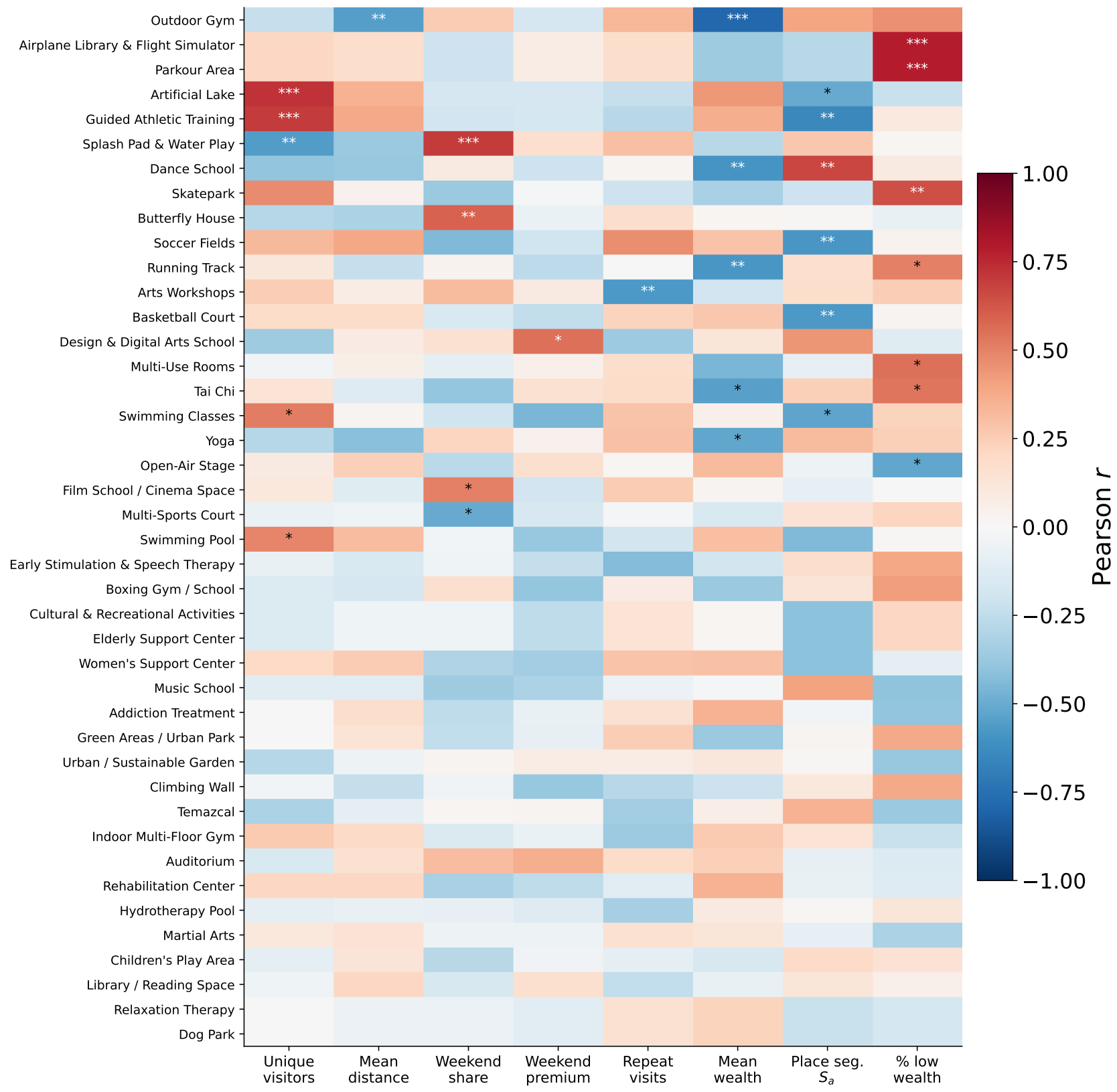

Fig. S4 Specific-activity presence against visit outcomes. Pearson correlations between the presence of each individual activity and the visit outcomes. Rows ordered by their strongest association (** $p < 0.05$, * $p < 0.10$).

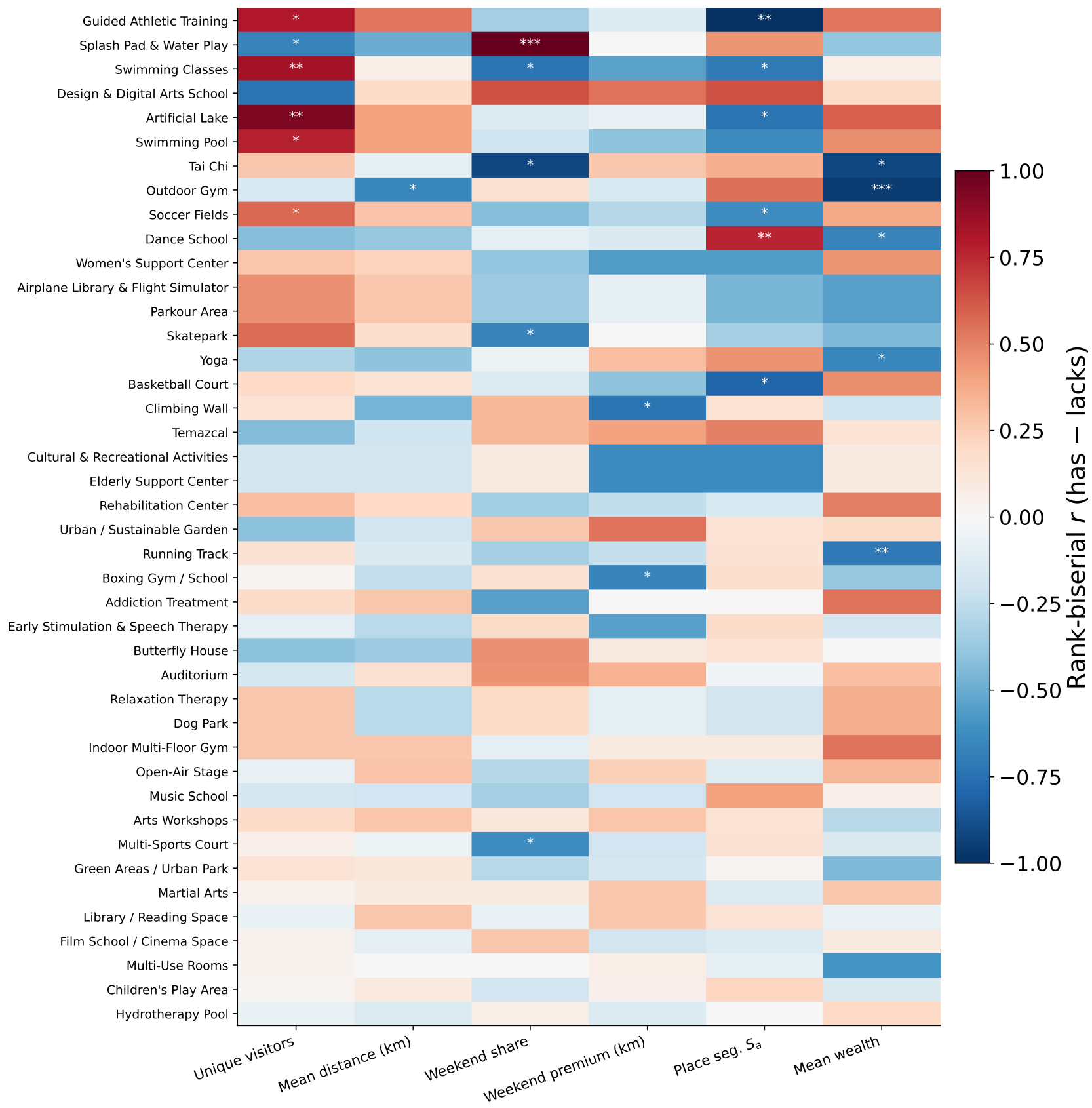

Fig. S5 Effect of offering each individual service. Rank-biserial effect sizes comparing the visit outcomes of sites that offer each activity against those that do not (positive values: sites with the activity score higher). Rows ordered by mean absolute effect (** $p < 0.05$, * $p < 0.10$).

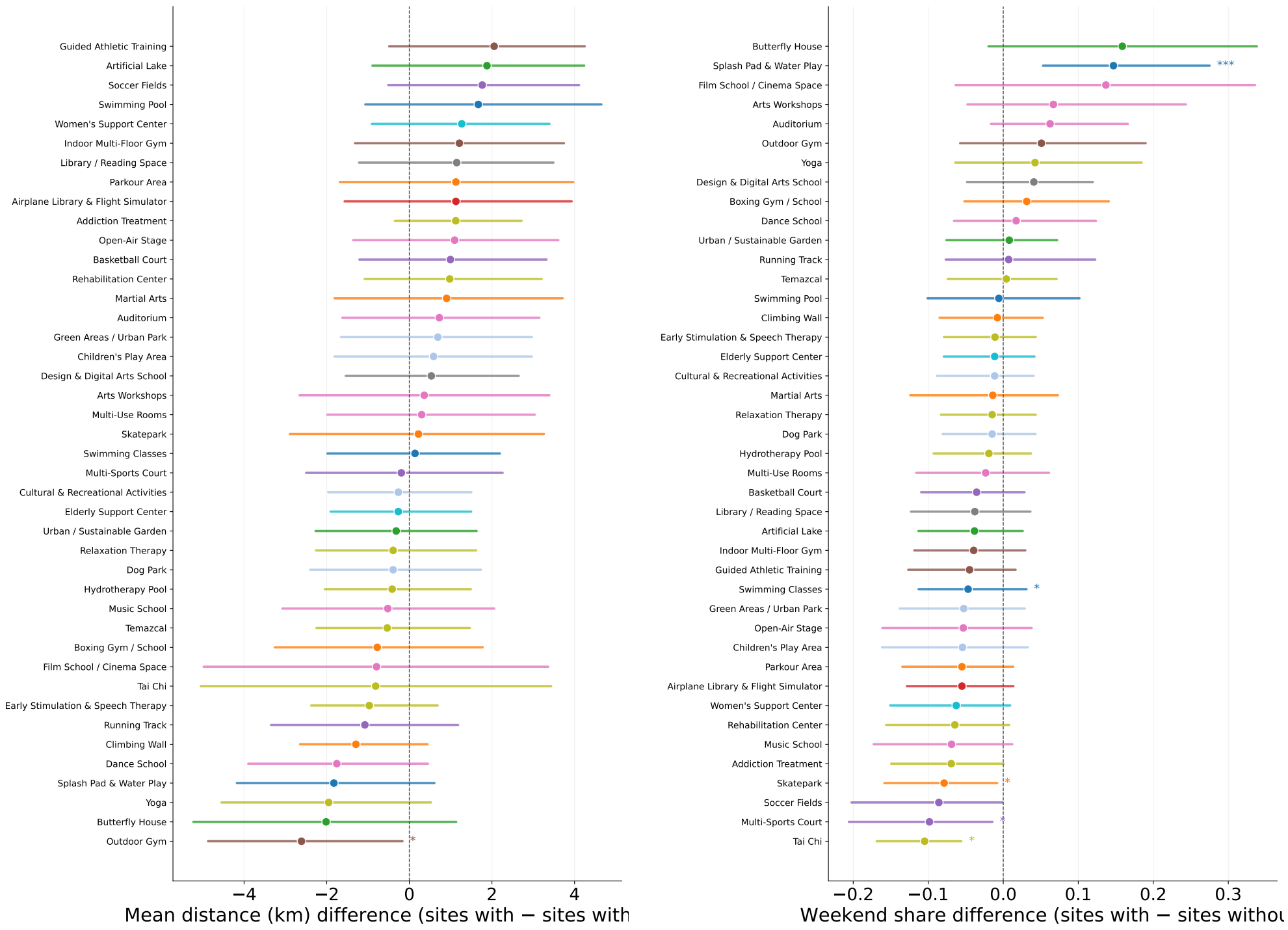

Fig. S6 Effect of each service on catchment distance and weekend use. Difference in mean distance from home to site (left) and weekend share (right) between sites that offer each activity and those that do not, with bootstrap 95% confidence intervals, colored by service domain (** $p < 0.05$, * $p < 0.10$).

S5 Public transport accessibility

The transit score of the main text combines five quantities measured within each site's one-kilometer buffer: the route count and the volume of weekday trips (each on a log scale), the presence of a metro stop, the presence of a BRT stop, and the number of modes, with fixed weights 0.30, 0.30, 0.20, 0.10 and 0.10. The route count and the weekday-trip volume are the two quantities that carry the association with visit volume, and each tracks unique visitors on its own ($r = 0.73$ and $r = 0.72$ on the log scale), while the mode count and the metro and BRT indicators are weaker in isolation ($r = 0.37$, 0.33 and 0.29). Recomputing the score under alternative weightings leaves its association with visit volume between $r = 0.56$ and $r = 0.75$, significant throughout, and an unsupervised first principal component of the five inputs gives $r = 0.62$ (Table S4).

Table S4 Correlation between the transit score and unique visitors (log scale) under alternative weightings of its five inputs, and the correlation of each input on its own. Weights are listed in the order (routes, weekday trips, metro, BRT, modes). Pearson r with two-sided permutation p, thirteen sites.

Weighting	(routes, trips, metro, BRT, modes)	r	p
Main text	(0.30, 0.30, 0.20, 0.10, 0.10)	+0.64	0.019
Equal	(0.20, 0.20, 0.20, 0.20, 0.20)	+0.56	0.045
Rail-heavy	(0.25, 0.25, 0.30, 0.10, 0.10)	+0.58	0.038
Supply only	(0.33, 0.33, 0, 0, 0.33)	+0.69	0.008
Routes and trips only	(0.50, 0.50, 0, 0, 0)	+0.75	0.003
First principal component	unsupervised	+0.62	0.026
Each input on its own			
Route count		+0.73	0.004
Weekday trips		+0.72	0.005
Number of modes		+0.37	0.220
Metro present		+0.33	0.282
BRT present		+0.29	0.329

Table S5 Per-utopia transit profile within the one-kilometer perimeter buffer, sorted by transit score.

Utopía	Stops	Routes	Modes	Wkday trips	Metro	BRT	Transit
Olini	237	115	5	162,784	yes	yes	0.94
Teotongo	227	152	6	159,342	yes	no	0.90
Tezontli	173	45	6	76,857	yes	yes	0.76
Ixtapalcalli	212	55	4	102,412	yes	no	0.66
Cuauhtlicalli	87	22	5	28,344	yes	yes	0.53
Atzintli	228	37	5	78,880	no	no	0.41
Meyehualco	203	41	4	79,010	no	no	0.40
La Cascada	95	53	2	58,009	no	no	0.35
Papalotl	172	32	4	50,502	no	no	0.32
Quetzalcoatl	192	26	4	63,121	no	no	0.31
Libertad	137	25	3	36,474	no	no	0.23
Barco	93	22	3	31,032	no	no	0.19
Tecoloxtitlan	73	16	3	9,144	no	no	0.03

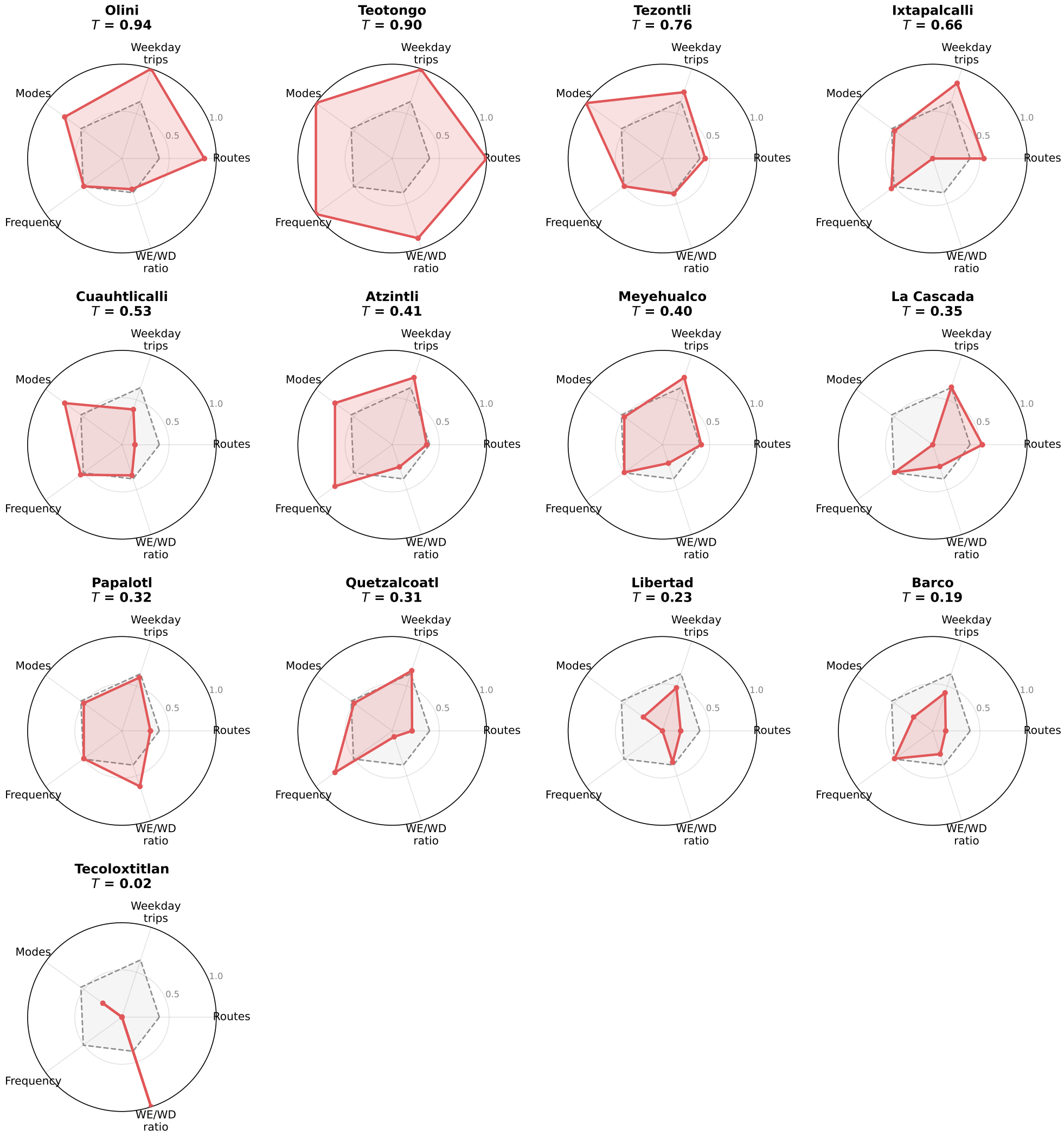

Fig. S7 Transit profile of each Utopía, resolving the transit score into its within-buffer sub-metrics: the route count, weekday-trip volume, number of modes, service frequency (the negative of peak headway) and the ratio of weekend to weekday trips, each rescaled to run from zero to one across the thirteen sites. Each panel shows one site (solid) against the network mean (dashed), ordered by the transit score T.

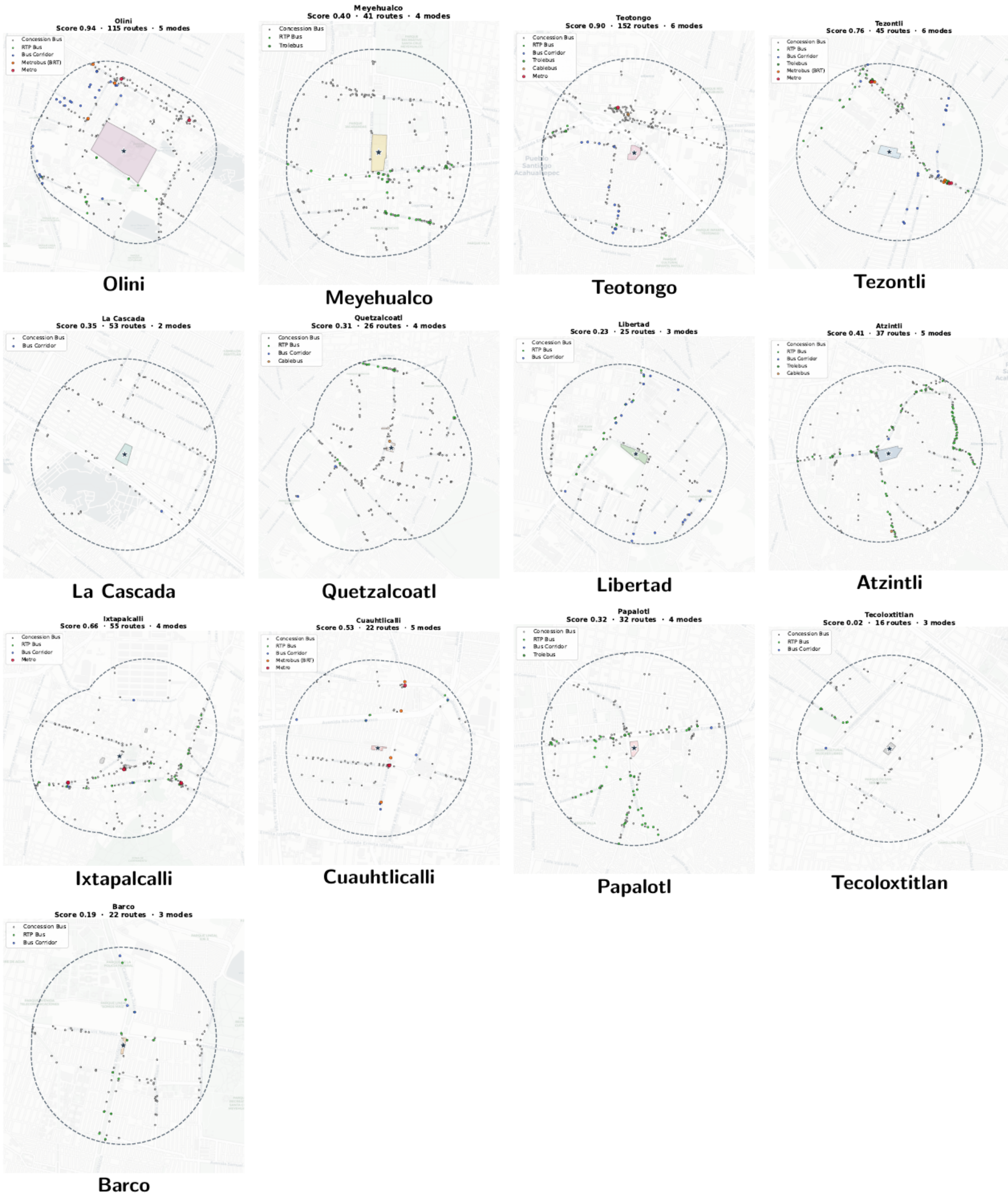

Fig. S8 Public transport within one kilometer of each site. Stops within the one-kilometer perimeter buffer of each Utopía, colored by mode.

S6 Surrounding crime

The Fiscalía General de Justicia open data portal lists incidents at the offense-string level, which is too granular for cross-site comparison. We collapse the offense strings into fifteen analytic categories using a hand-encoded mapping that we validate against the registry's own `categoria_delito` field. The non-criminal residual (`HECHO NO DELICTIVO`) is excluded from all per-utopia analyses, although we keep it during the time-series construction as a sanity check on reporting volume. For the per-category correlation analysis we keep only categories with a non-zero count at three or more sites.

Table S6 Per-utopia crime profile within the one-kilometer perimeter buffer (combined 2023 and 2024), sorted by crime density. The violent share is the fraction of incidents in violent categories (homicide, firearm injury, kidnapping, rape and the violent variants of robbery).

Utopía	Total crimes	Crimes per km^2	Violent crimes	Violent share
Tezontli	2,355	606	95	0.040
Libertad	2,381	587	77	0.032
Atzintli	2,208	551	104	0.047
Olini	3,593	528	104	0.029
Ixtapalcalli	2,694	514	93	0.035
Papalotl	1,787	494	78	0.044
Quetzalcoatl	2,816	486	130	0.046
Cuauhtlicalli	1,631	457	36	0.022
Barco	1,508	420	41	0.027
Meyehualco	1,829	418	66	0.036
Tecoloxtitlan	1,260	354	63	0.050
La Cascada	1,162	301	43	0.037
Teotongo	1,070	290	39	0.036

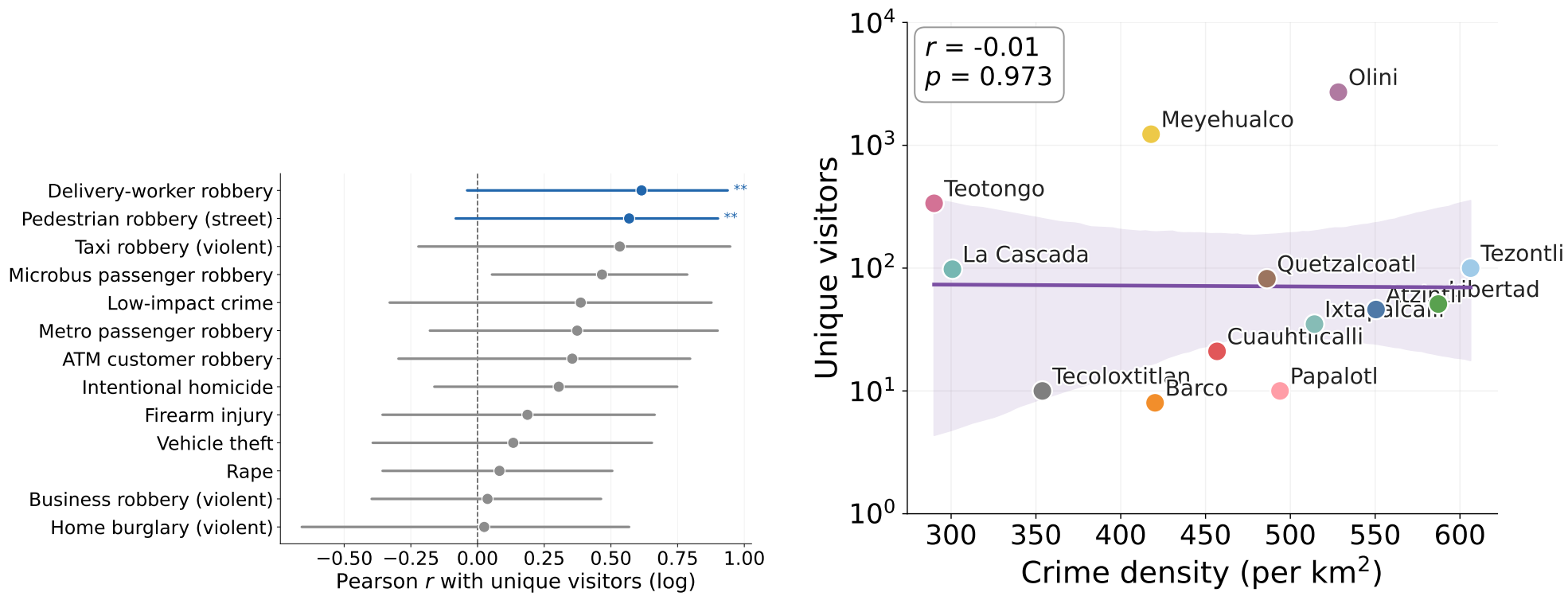

Fig. S9 Crime volume against visit volume. Per-category correlation between within-buffer crime counts and unique visitors (left) and crime density against unique visitors (right, $r = -0.01$).

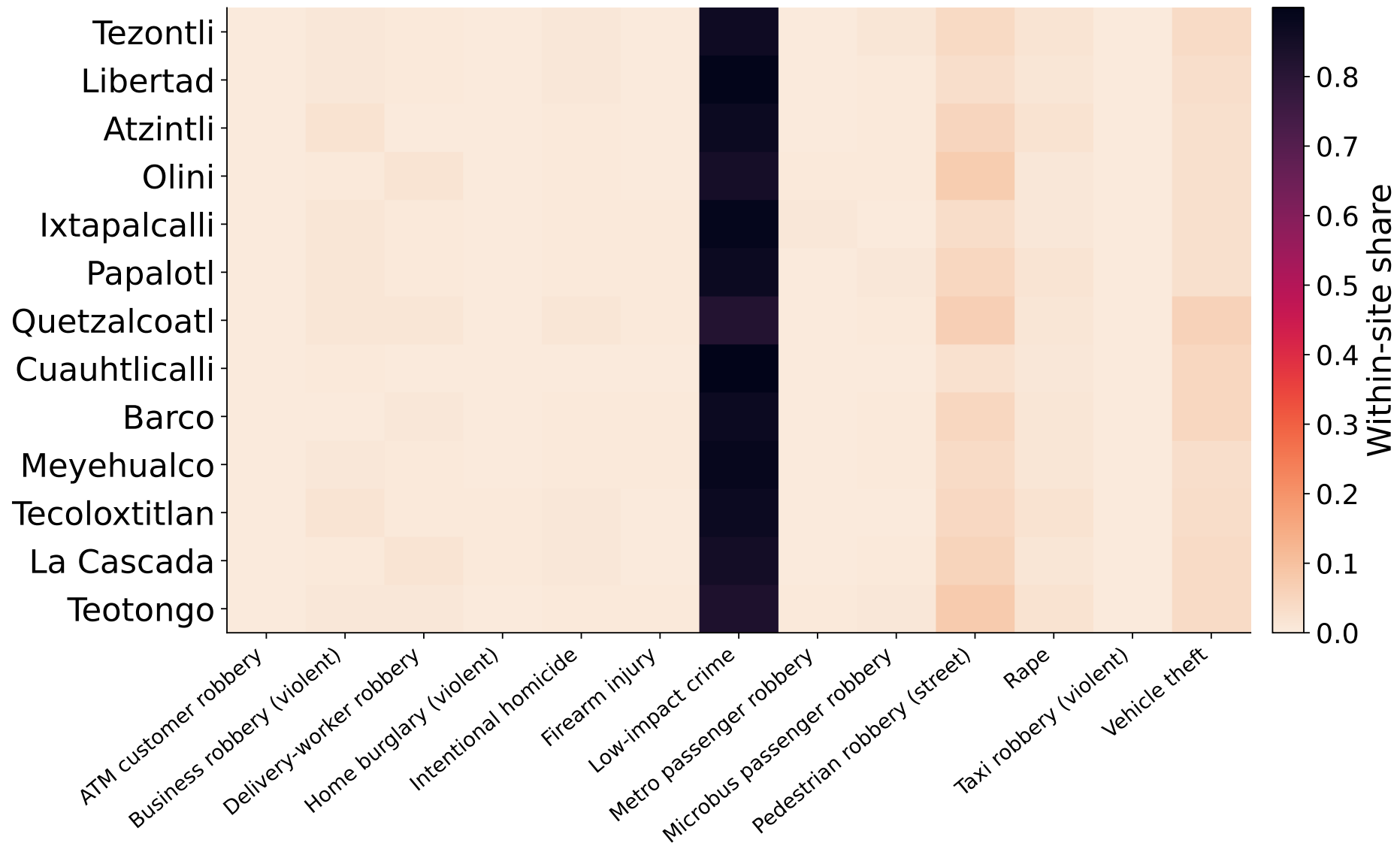

Fig. S10 Within-site crime category mix. Share of each crime category within the one-kilometer buffer of every site (rows normalized to one), ordered by crime density.

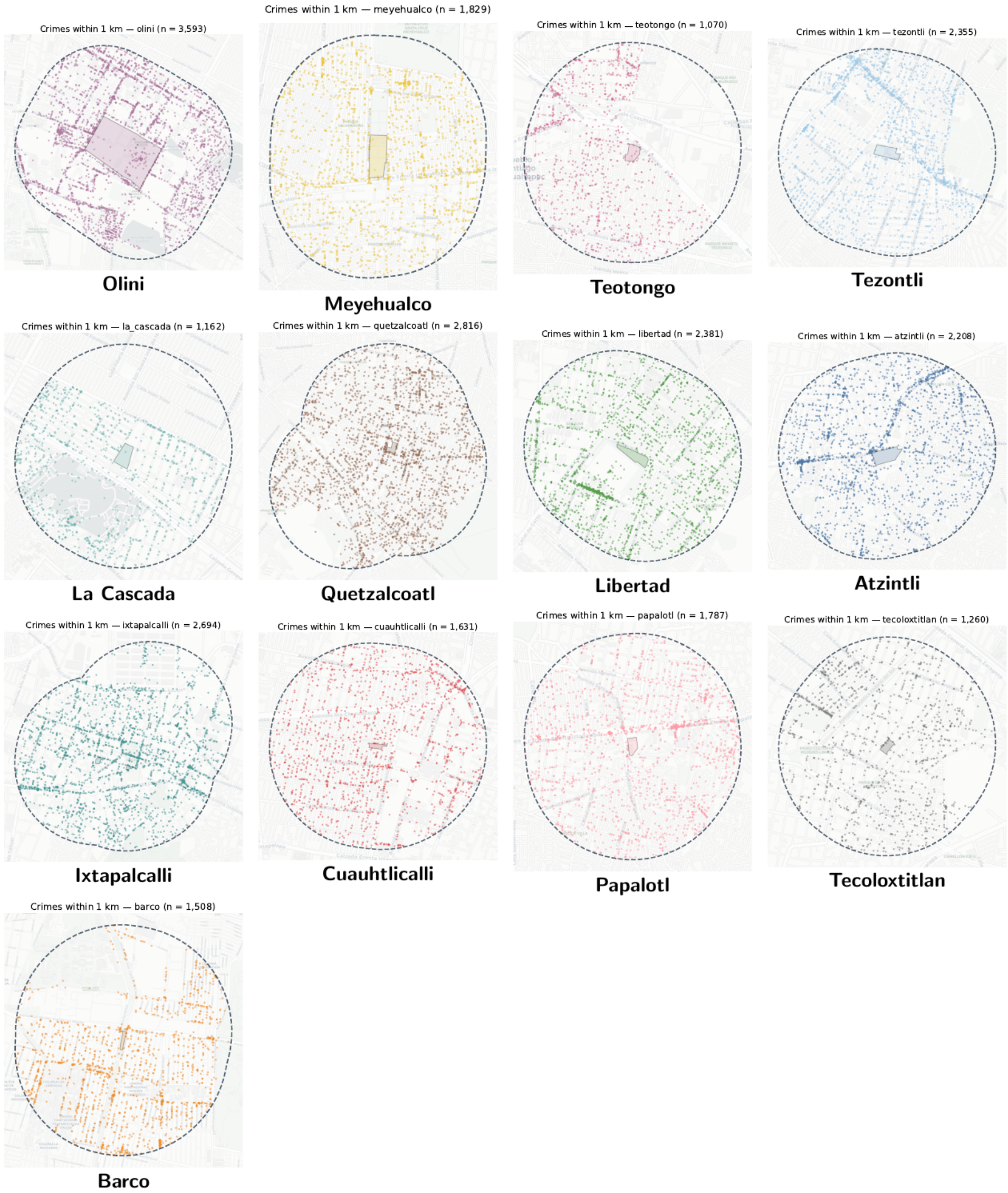

Fig. S11 Reported crime within one kilometer of each site. Incident locations within the one-kilometer perimeter buffer of each Utopía (combined 2023–2024).

S7 The combined accessibility index

The composite combines three z-scored axes. The crime axis is the mean of the standardized crime density and the standardized violent share; the transit axis is the standardized transit score, which already integrates routes, weekday trips, metro, BRT and modes; the services axis is the mean of the standardized distinctiveness index and the standardized number of facilities. The default composite is the unweighted mean (services plus transit minus crime). We also report three sensitivity schemes:

- Crime-heavy: $w_C = 0.5$, $w_T = 0.25$, $w_S = 0.25$.
- Transit-heavy: $w_C = 0.25$, $w_T = 0.5$, $w_S = 0.25$.
- Services-heavy: $w_C = 0.25$, $w_T = 0.25$, $w_S = 0.5$.

Figure S12 shows how the site ranking changes under each of these schemes, and Figure S13 splits each site's composite into the contribution of its three axes.

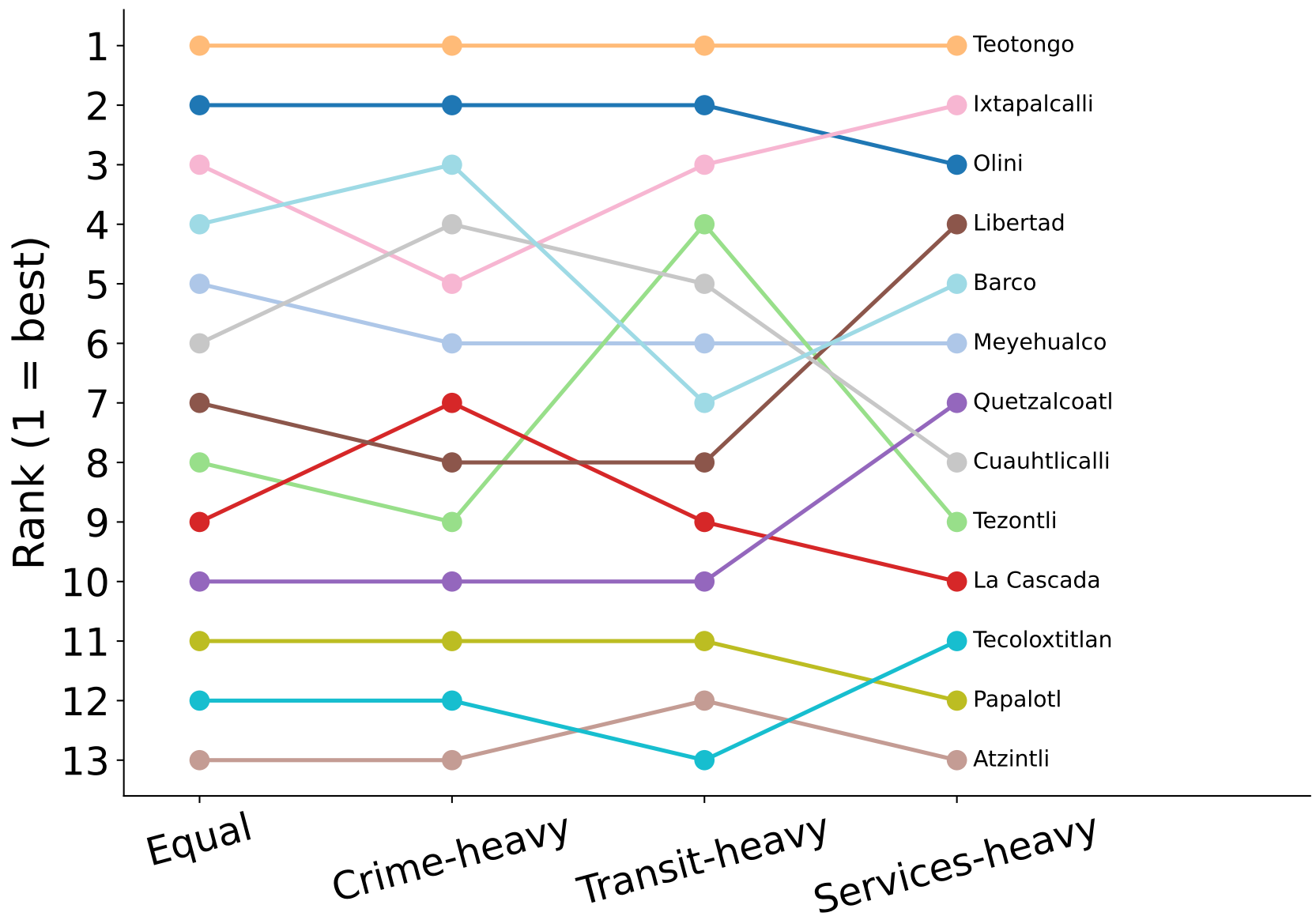

Fig. S12 Composite ranking under the four weight schemes (equal, crime-heavy, transit-heavy, services-heavy). Each line traces one site's rank across the schemes.

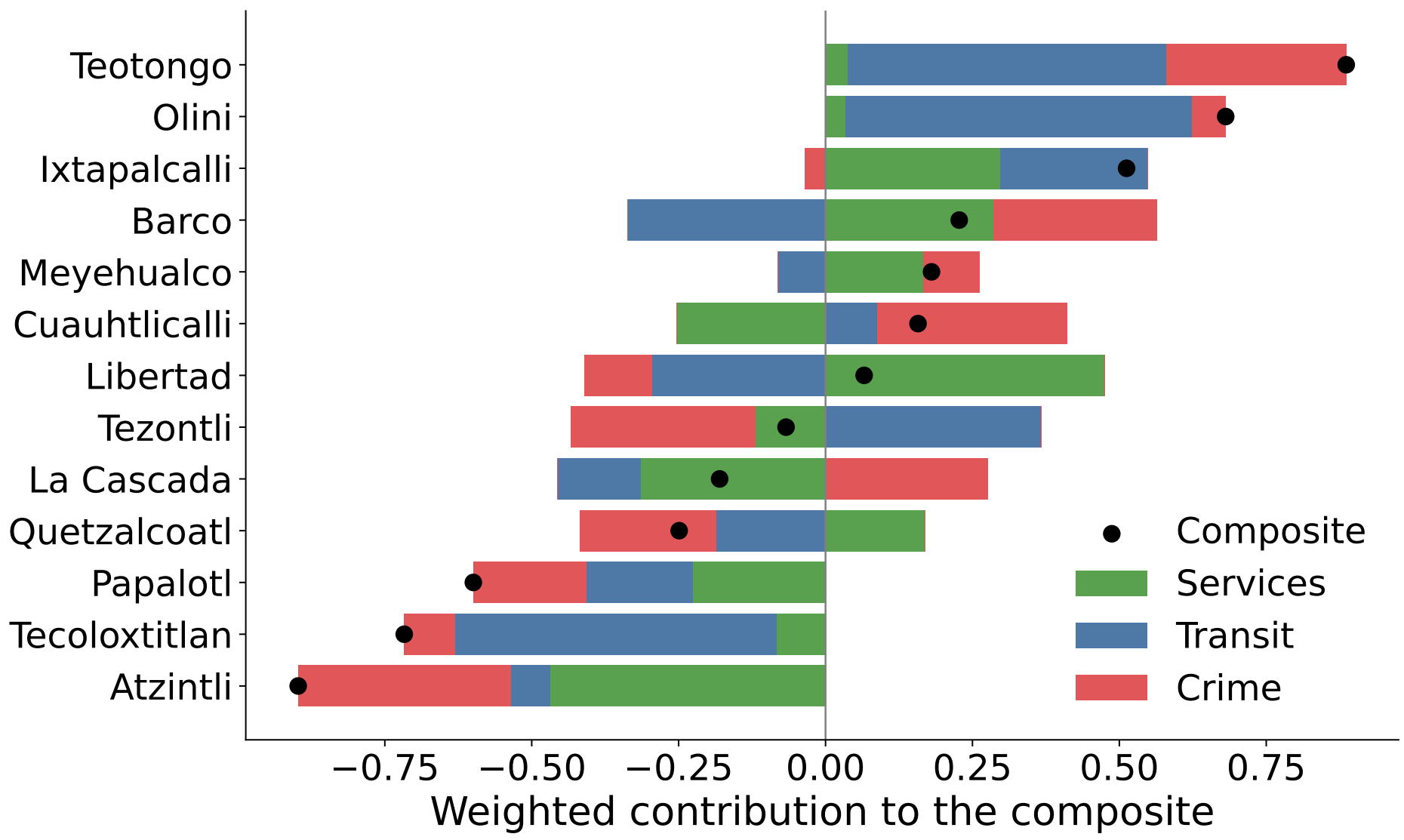

Fig. S13 Composite decomposition by axis. Each bar splits into the contribution of services, transit and the negated crime axis, with the resulting composite marked.

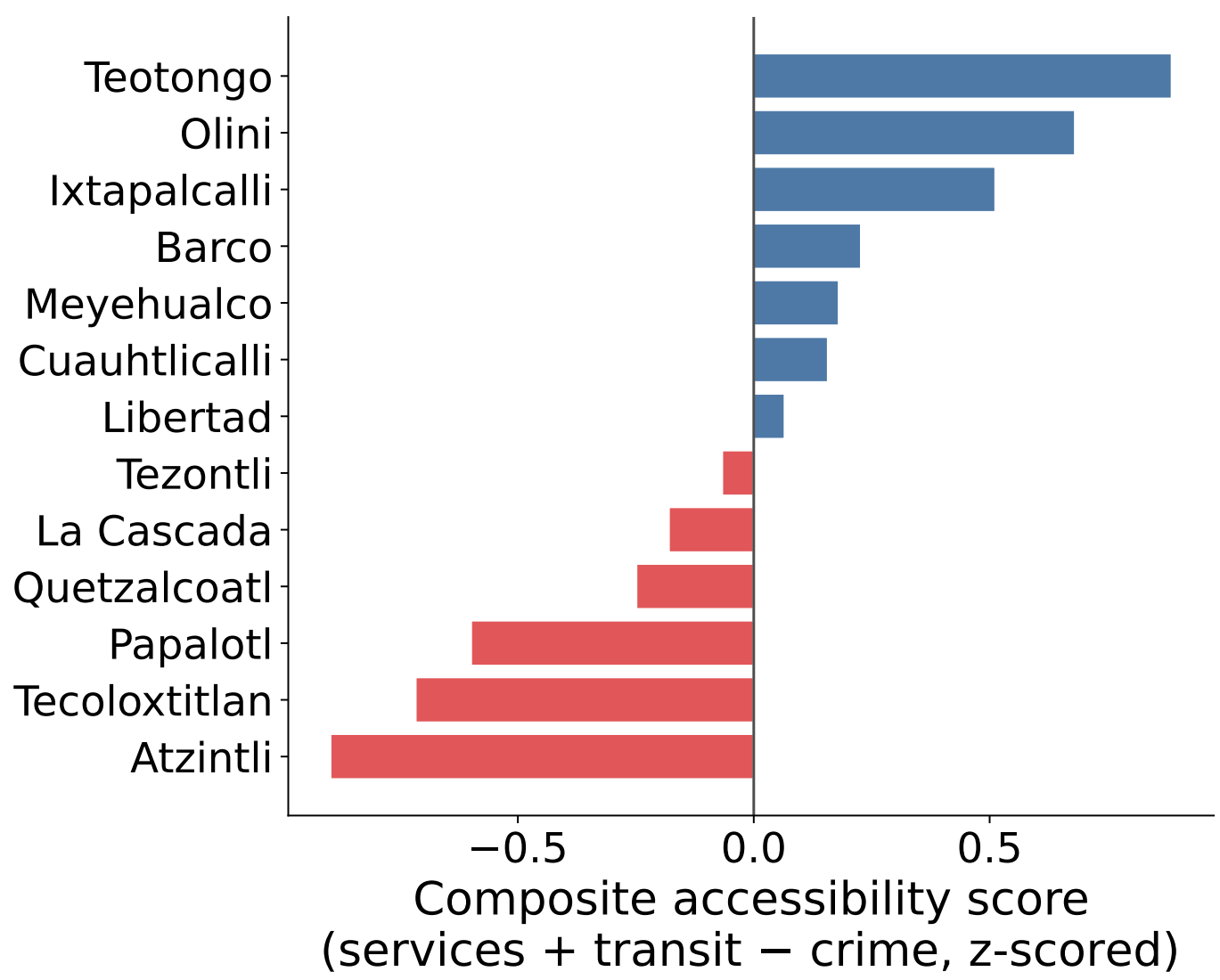

Fig. S14 Composite accessibility score per site (services plus transit minus crime, equal weights, z-scored), ordered from highest to lowest.

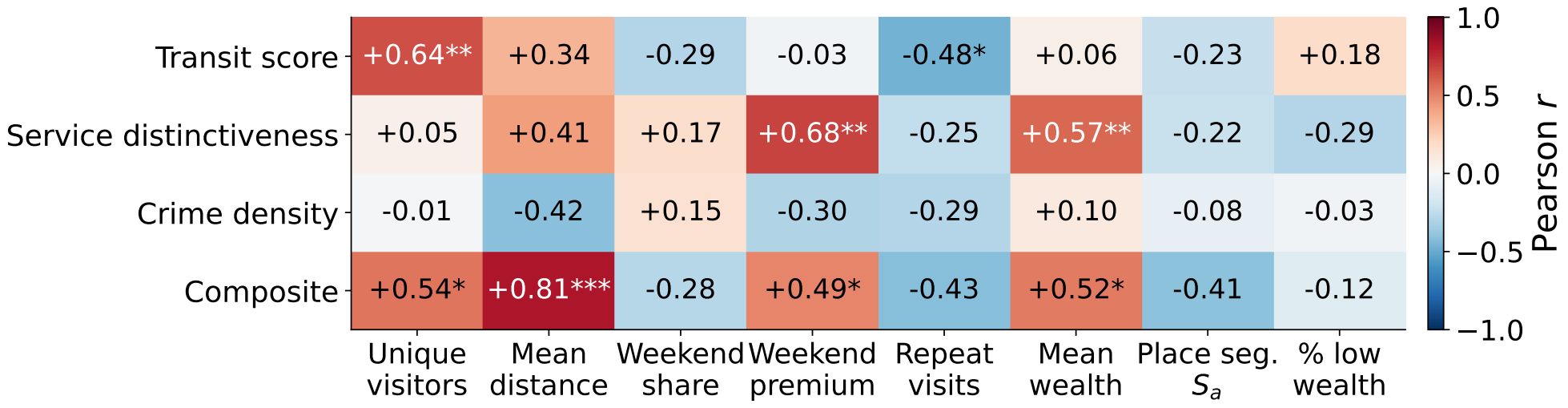

Fig. S15 The three site axes and the composite against the full set of visit outcomes. Pearson correlations between the transit score, the service distinctiveness index, crime density and the equal-weight composite and all eight per-site outcomes, the complete matrix behind Figure 7a of the main text. Cells marked ** $p < 0.05$, * $p < 0.10$.

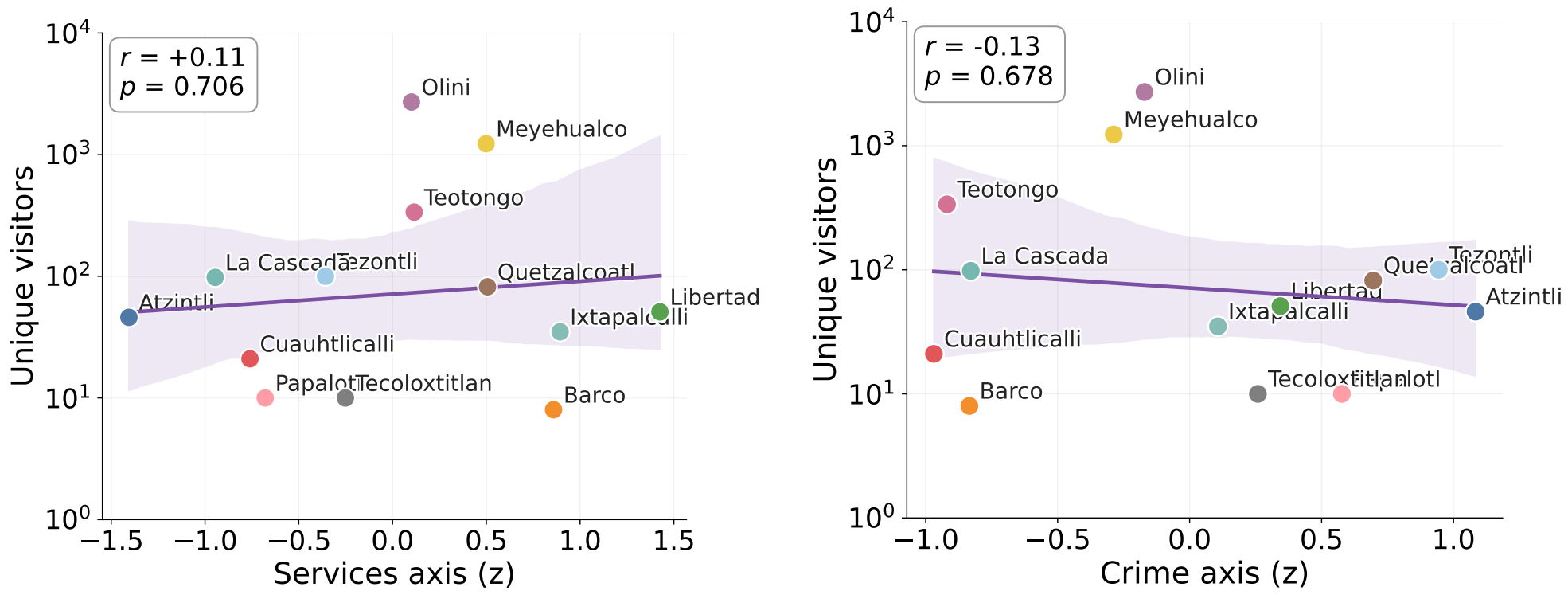

Fig. S16 The services and crime axes against unique visitors. The services axis (left) and the crime axis (right) against unique visitors on a log scale, each with a least-squares fit and its bootstrap 95% confidence band.

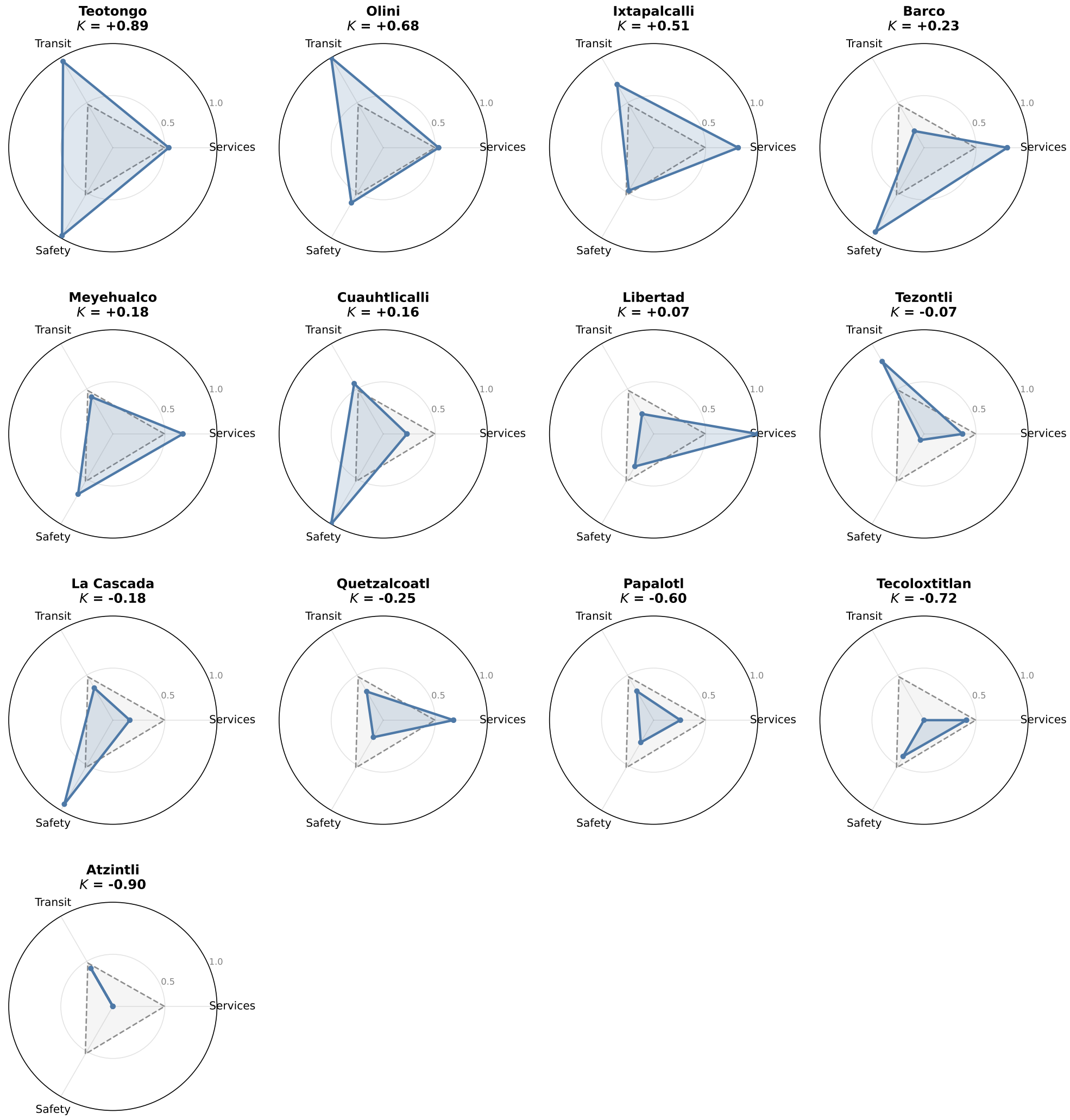

Fig. S17 Three-axis profile of each Utopía (services, transit and safety, where safety is one minus the normalized crime axis, each on a scale from zero to one). Each panel shows one site (solid) against the network mean (dashed), ordered by the composite score K.

S8 Temporal dynamics

The daily series of unique visitors is reindexed to a regular daily frequency over the study window and decomposed with STL [2] using a weekly period (= 7). The trend component carries almost all of the variation; the weekly seasonal component is small in amplitude (under one visitor per day separating the average weekend from the average weekday).

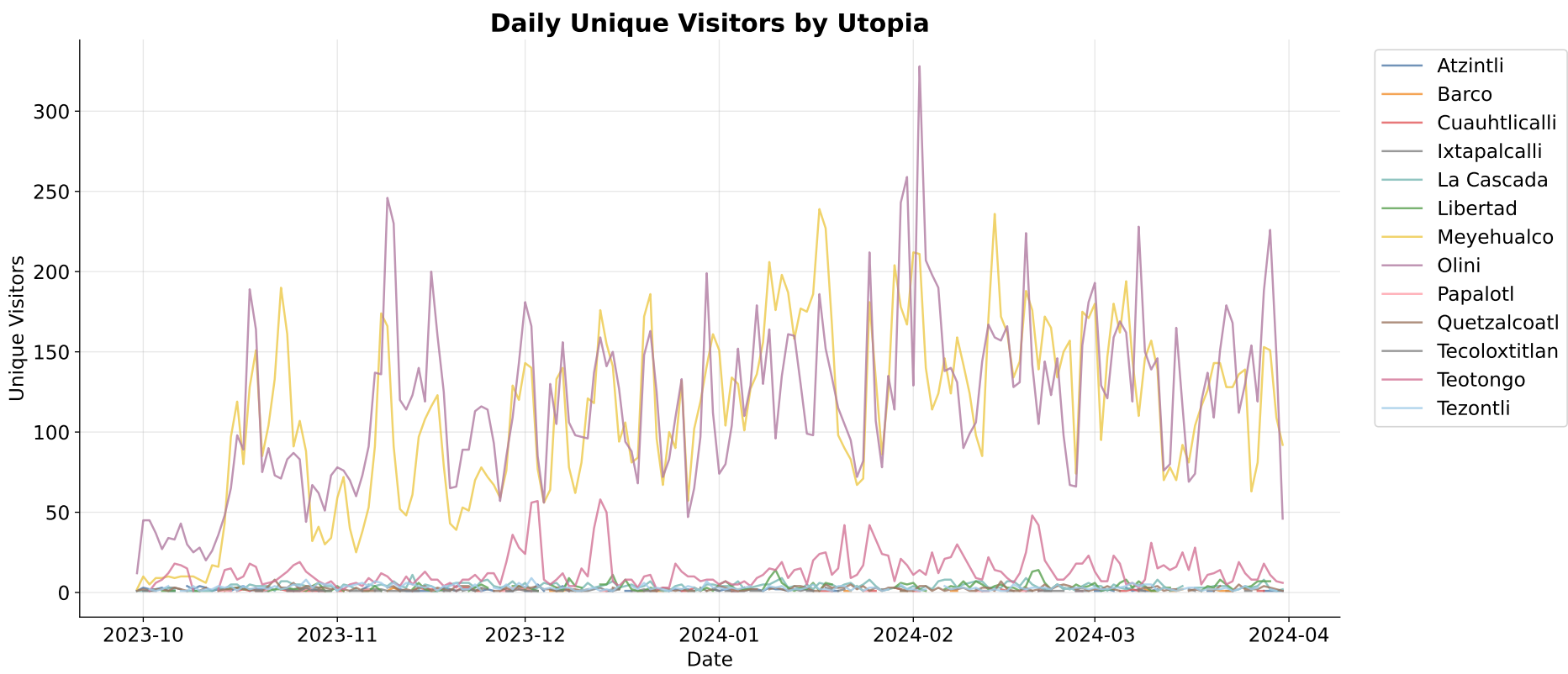

Fig. S18 Daily unique visitors by site. Daily unique-visitor series for each Utopía across the study window.

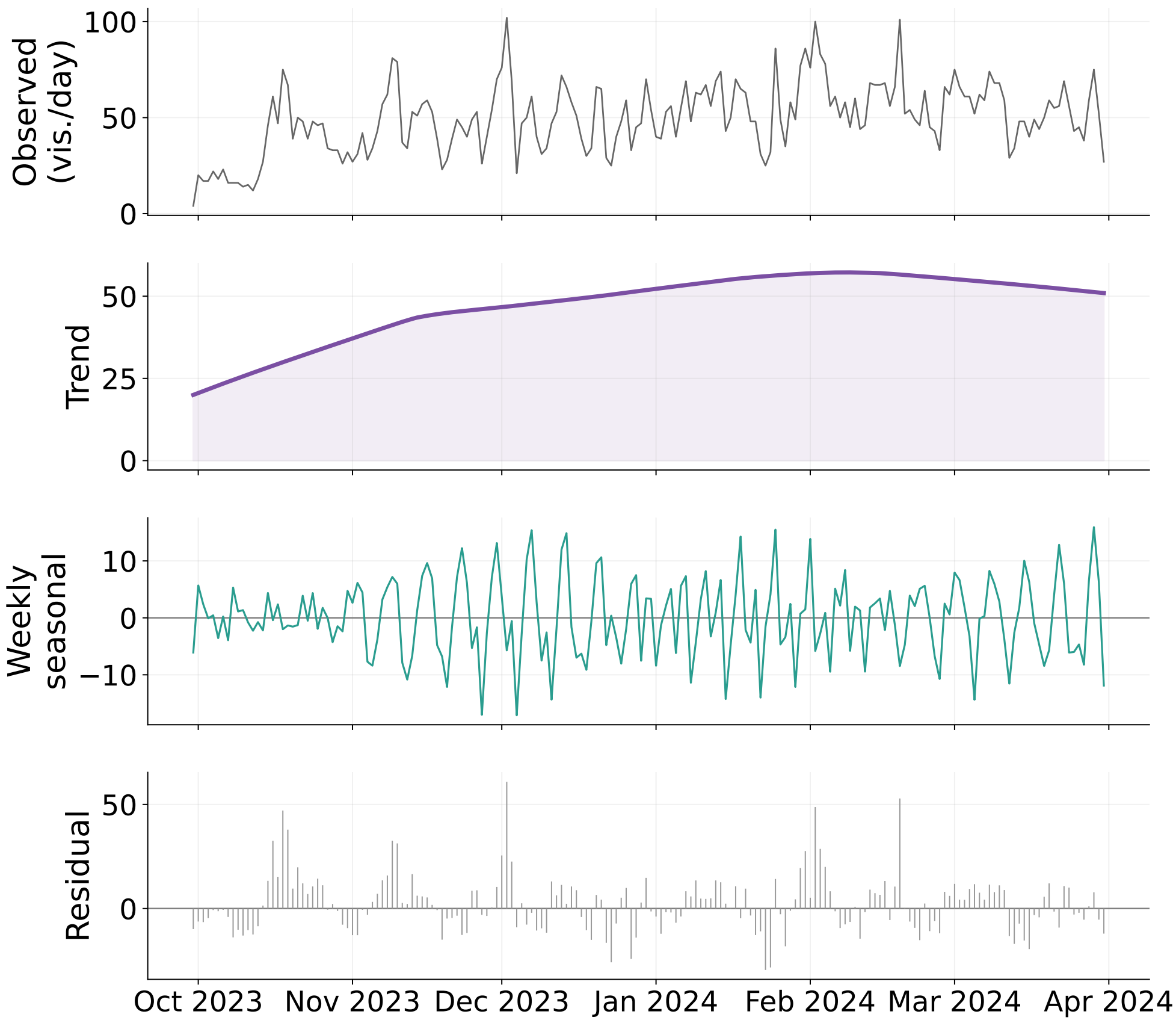

Fig. S19 STL decomposition of the network-wide daily visitor series into observed, trend, weekly seasonal and residual components (weekly period) [2].

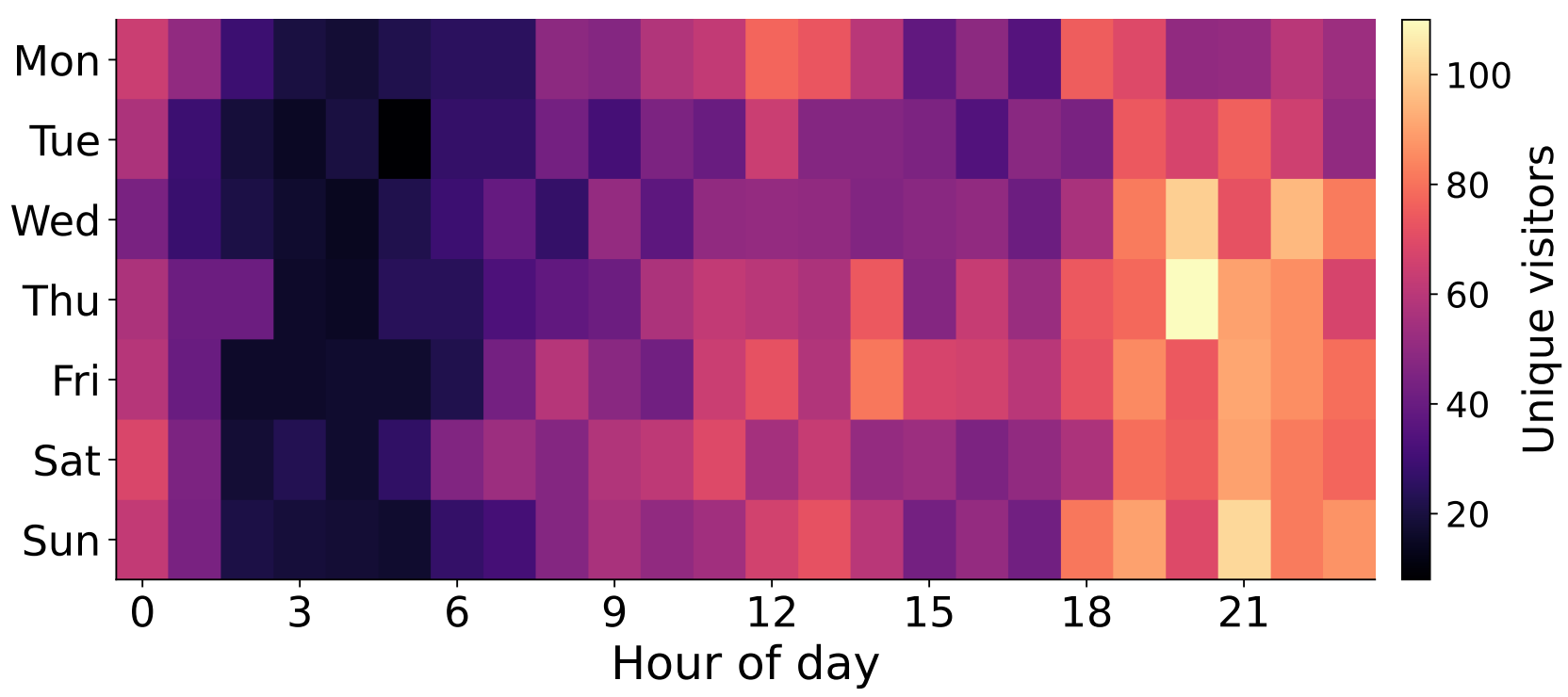

Fig. S20 Activity by day of week and hour of day. Unique visitors by weekday (rows) and hour of day (columns).

S9 Visitor origins

Table S7 lists the boroughs and municipalities that send the most visitors to the network, pooled across all thirteen sites, behind the aggregate origin shares given in the main text.

Table S7 The twelve largest boroughs of origin for visitors to the network, by unique visitors with a detected home location pooled across all sites.

Borough / municipality	Entity	Unique visitors	Share (%)
Iztapalapa	Mexico City	2,110	44.9
Coyoacán	Mexico City	333	7.1
Nezahualcóyotl	State of Mexico	289	6.1
Cuauhtémoc	Mexico City	194	4.1
Iztacalco	Mexico City	187	4.0
Tlalpan	Mexico City	179	3.8
Benito Juárez	Mexico City	138	2.9
Álvaro Obregón	Mexico City	123	2.6
Xochimilco	Mexico City	91	1.9
Tláhuac	Mexico City	91	1.9
Gustavo A. Madero	Mexico City	82	1.7
Miguel Hidalgo	Mexico City	81	1.7

S10 Data and code availability

The aggregated per-site metrics behind every table and figure in the main text and in this document, together with the analysis code that produces them, are kept in the project repository at https://github.com/ollin18/utopias. The pipeline that turns raw location pings into stops, activity clusters and home locations (Sections 2.2 and 2.3 of the main text) is released separately as `gc-lbs`, available at https://github.com/ollin18/gc-lbs and archived on Zenodo [3]. The raw LBS visit records cannot be redistributed because of the privacy terms under which they were licensed; they can be requested from the original data provider under a data-use agreement, and enquiries may be directed to the corresponding author. The GTFS feed of the Mexico City public transport network and the crime registry of the Fiscalía General de Justicia are open data, published on the city's open data portal (https://datos.cdmx.gob.mx).

References

1. Moro, E., Calacci, D., Dong, X. & Pentland, A. Mobility patterns are associated with experienced income segregation in large US cities. *Nature Communications* **12,** 4633 (2021).
2. Cleveland, R. B., Cleveland, W. S., McRae, J. E. & Terpenning, I. STL: A Seasonal-Trend Decomposition Procedure Based on Loess. *Journal of Official Statistics* **6,** 3–73 (1990).
3. Langle-Chimal, O. D. *gc-lbs: a processing pipeline for location-based services data* Software, version v0.1.0-alpha, https://github.com/ollin18/gc-lbs. 2026.
